# Design, modeling and cost analysis of 8.79 MW solar photovoltaic power plant at National University of Sciences and Technology (NUST), Islamabad, Pakistan

**DOI:** 10.1038/s41598-024-74187-w

**Published:** 2024-10-25

**Authors:** Shabahat Hasnain Qamar, Dawid Piotr Hanak, Majid Ali, Joao Gomes, Khalid Zia Khan

**Affiliations:** 1 Department of Mechanical Engineering, National University of Sciences and Technology (NUST), Islamabad, Pakistan; 2 Net Zero Industry Innovation Centre, Teesside University, Ferrous Road, Middlesbrough, TS2 1DJ UK; 3 U.S.-PAKISTAN Center for Advanced Studies in Energy (USPCASE), National University of Sciences and Technology (NUST), Islamabad, Pakistan; 4 University of Gävle, Gävle, Sweden

**Keywords:** Photovoltaic, Photovoltaic power plant, PV syst software, Cost analysis, Climate-change impacts, Photovoltaics

## Abstract

Climate change, as a critical global concern, has fueled our efforts to address it through different strategies. In response to the critical worldwide issue of climate change, we suggested a Photovoltaic (PV) system at the National University of Sciences and Technology (NUST) in Islamabad, Pakistan (latitude: 33.724530 N, longitude: 73.046869, terrain elevation: 552 m). Islamabad is located in a region blessed with enormous solar resources, boasting a daily horizontal solar irradiance of 1503.45 kWh/m^2^ and an average daily solar irradiance of 5.89 kWh/m^2^, with an exceptional solar fraction of 98.99%. The ambient air temperature, averaging 23.21 °C, reaches its maximum in June and its minimum in December. Our research thoroughly evaluates the system’s performance, accounting for various losses and utilizing modern PVsyst software. Over the course of 18 years, our PV system is expected to save 75,478.60 tons of CO_2_, the equivalent of planting 348,754 teak trees. Furthermore, the cost of energy generation is an affordable 0.0141 US $/kWh, much lower than traditional rates, including the Sherif cost of 0.028$/kWh. Along with the performance research, we conducted a detailed cost analysis, projecting the starting cost and cash flow, and discovered that the plant would be in surplus within 12 years of installation. Our system is positioned to generate 11,270,771 kWh/year with a respectable performance ratio (PR) of 76.2% and a Capacity Utilization Factor (CUF) of 16%. Our findings not only highlight the potential of renewable energy but also provide important insights for future sustainable energy programs.

## Introduction

**Fig. 1:**
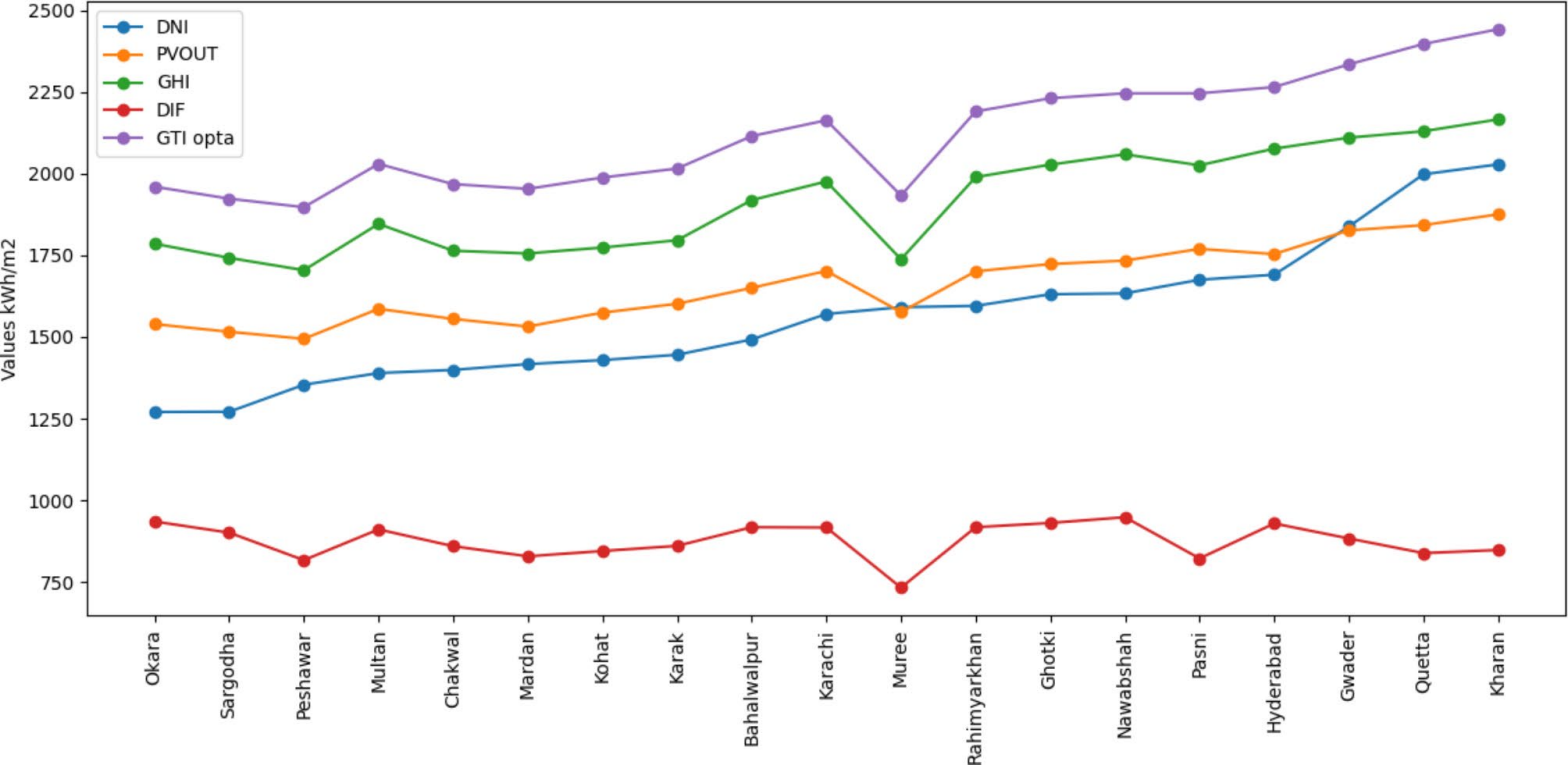
Solar resource variability in different regions of Pakistan.

**Fig. 2:**
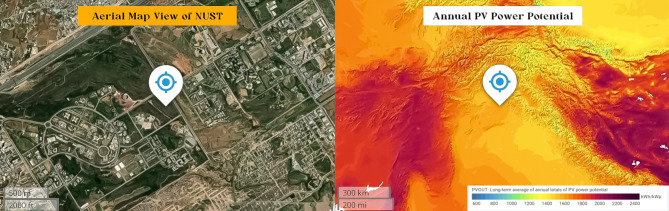
(**a**) Aerial map of NUST. (**b**) Solar PV power potential. (Source: Google Maps, 2024. Used under principles of fair use for educational and non-commercial purposes. Available from: https://www.google.com/maps).

Energy is a major driver of modern economies, essential for education, healthcare, agriculture, and employment, and serves as a pillar of a country’s economic viability. Pakistan, a growing country covering 803,950 km^2^ and ranking sixth in the world in terms of population, faces a severe energy crisis^1^. Only 62% of the population has grid access, resulting in a per capita electricity supply of only 520 kWh^2^. Notably, around 58% of the country’s people live in distant rural areas that are largely disconnected from the grid. This energy shortage, reaching 9–15 GW, has a significant impact on the nation’s businesses and causes severe blackouts and load shedding, with an annual economic cost of $3.8 billion^3,4^. Pakistan has a varied spectrum of renewable energy resources, including hydro, solar, wind, and biomass, to address these difficulties^5^. Importantly, Pakistan is rated as one of the top 20 most appealing countries in the world for renewable investment but in recent years, Pakistan has seen an increasing imbalance between electricity demand and supply, which is most noticeable during the hot summer months. This imbalance has resulted in widespread power outages, with cities seeing up to 10–12 h of blackout and rural areas facing even longer outages of 16–18 h^6^. Pakistan relies substantially on thermal power, accounting for 68% of its total electricity generation capacity of 183,540 Gigawatt hours (GWh)^7^. Notably, the country lacks proven oil, coal, and natural gas deposits, as well as domestic power production capacity. This critical energy imbalance is highlighted by an average daily electricity gap of 4.7 million kWh, which can reach 7.5 million kWh on hot summer days^8^. In response, the Pakistan government is actively advocating for a transition to sustainable and renewable energy sources, such as wind and solar power, in order to address acute electricity shortages. Currently, renewable energy accounts for a small portion of Pakistan’s overall energy mix, with the country relying heavily on fossil fuels to cover its energy needs. This reliance on fossil fuels not only puts a tremendous strain on the national economy, but also creates a number of environmental issues, such as the greenhouse effect, CO_2_ emissions, global warming, and unpredictable weather patterns. Furthermore, the continued use of fossil fuels depletes natural resources at an alarming rate. As a result, there is an urgent need to develop a new energy economy in which renewable sources such as wind, solar, and biomass take center stage. This transformation not only offers to minimize the import expense linked with fossil fuels, but it also promises to address the current climate-related concerns. Pakistan’s energy problem is mostly caused by the country’s dependency on thermal resources such as coal, oil, and natural gas, all of which are not only expensive but also in limited supply^9^. Furthermore, hydroelectricity, which was formerly a considerable source, has recently declined in importance. Renewable energy sources, on the other hand, currently account for only 0.3% of the country’s energy needs, a fraction too small to relieve the situation^10^. Solar energy emerges as a powerful solution to these difficulties among the diversity of renewable energy options^11^. Globally, it has positioned itself as the most cost-effective technology, with the ability to transform Pakistan’s energy landscape. Large-scale solar photovoltaic and wind turbine projects have assumed precedence in Pakistan’s Sustainable Action Plan 2009^12^, which was amended in 2013, owing to falling technology costs. These semiconductors incorporated within solar modules provide a dependable, maintenance-free, and environmentally beneficial source of energy that is adaptable to changing climatic circumstances. In the following parts, we will go deeper into the literature, investigating the history and possibilities of solar energy in Pakistan, and charting a course toward a more sustainable and energy-rich future. Figure 1 depicts a combined line graph illustrating key solar parameters for various locations in Pakistan (Direct Normal Irradiation—DNI, Specific Photovoltaic Power Output—PVOUT, Global Horizontal Irradiation—GHI, Diffuse Horizontal Irradiation—DIF, and Global Tilted Irradiation at Optimum Angle—GTI opta). The data was sorted in ascending order based on DNI values, allowing for a clear comparison of solar resource potential across different locations. Kharan has the greatest GTI opta rating among these places, while simultaneously having the best DNI, GHI, and PVOUT value, indicating ideal conditions for solar energy generation. Nawabshah has the greatest DIF. This graph depicts how these factors vary among places, highlighting the specific regions in Pakistan with the greatest solar resource potential. Figure 2(a)) depicts an aerial map of Islamabad’s National University of Sciences and Technology (NUST). Meanwhile, Fig. 2(b) depicts the solar PV potential map for this specific area. These maps demonstrate Islamabad’s enormous solar energy potential, making it a desirable place for electricity production via solar PV installations.

## Literature review and background

Pakistan’s electricity generation is mostly based on oil, gas, hydropower, and nuclear energy, which contribute 35.3%, 29.1%, 30%, and 5.5%, respectively, to total power production^13^. Despite considerable domestic coal deposits, the usage of coal is modest, accounting for barely 0.1% of the energy mix but since Pakistan has a large potential for solar energy, getting an annual average of 1900–2200 kWh/m^2^ of global solar radiation^14^. Pakistan’s steady solar radiation dispersion favors solar energy uses^15^. Various photovoltaic modules and system schemes have been investigated, making renewable energy system planning easier^16^. Techno-economic feasibility studies have been critical in examining the performance of low-cost renewable energy systems in Pakistan, particularly off-grid solutions for small loads^17^. Pakistan has an estimated solar energy reserve of up to 100,000 MW due to its ample sunshine^7^. Recognizing the potential of solar energy, the government prioritized the Quaid-e-Azam Solar Park project in Bahawalpur, Punjab. Pakistan updated its grid code and standard project documentation in March 2013 to encourage the development of solar power plants, offering incentives such as tax breaks and an 18.5% return on investment to entice investors^18^. These activities are in line with the country’s aim of reducing carbon emissions and managing electricity supply issues. Pakistan adopted legislation favoring hydropower, wind power, and photovoltaic energy to hasten the shift to renewable energy sources^19^. Collaboration with the US government and the World Bank has resulted in geographical solar energy and wind resource mapping studies that highlight Pakistan’s tremendous solar power potential, estimated at around 1600 GW, with certain places rivalling the world’s highest MENA region^20^. The need of well-maintained weather stations of accuracy was underlined in these investigations. Furthermore, talks on grid-connected PV power systems in rural communities, as well as the necessity for a realistic energy strategy, underscore Pakistan’s commitment to a sustainable energy future. Despite an increase in private thermal-based independent power plants, Pakistan’s power generation capacity was challenged by a financial crisis, resulting in a peak power shortfall of 4743 MW during the summer season^2,21^. This scene emphasizes the importance of continuing efforts to expand renewable energy and construct robust power infrastructure for long-term growth. According to the International Energy Agency (IEA), global solar PV capacity reached 402 gigatonnes (GW) at the end of 2017, with projections showing an additional development of nearly 580 GW, cementing its position as a frontrunner in renewable power capacity growth^22^. Akram et al.^23^ investigated the complexities of institutional and technical impediments to solar energy adoption in a separate study. Over the last two decades, the European Commission has invested heavily in solar energy research in order to reduce pollution, reduce carbon emissions, improve energy security, and diversify European nations’ energy portfolios. The difference-in-differences (DID) methodology was used by^24^ to examine the impact of emission trading systems (ETS) on economic growth and carbon emissions. Their research advocated carbon trading policies based on renewable energy projects. Akhtar et al.^25^ did a cost-saving comparison before and after the deployment of cost-saving certified emission reduction (CCER) systems. Over the last two decades, the European Commission has invested heavily in solar energy research in order to reduce pollution, reduce carbon emissions, improve energy security, and diversify European nations’ energy portfolios. Queseda et al.^26^ used the Sigma Plot optimization tool to estimate parameters in the logistic model, which performed better than linear models. Khatri et al.^27^ demonstrated that logistic modeling is a reliable tool for estimating and projecting data over long periods of time and has been applied in various domains. References^28–31^ used machine learning algorithms to measure global solar radiation and forecast diffuse solar radiation for the humid-subtropical climatic zone. Ur Rehman et al.^32^ designed an off-grid PV system for household users, demonstrating the diverse application of solar energy modeling and research. Raza et al.^33^ used Pakistan’s Logarithmic Mean Division Index to conduct a comprehensive analysis of CO_2_ emissions from electricity generation from 1978 to 2017. According to projections, the electricity industry alone will produce around 277.9 million metric tons of CO_2_ emissions by 2035.Grainger et al.^34^ calculated the baseline emission factor for the power production sector by considering characteristics such as annual fossil fuel consumption net efficiencies, annual energy outputs, and carbon emissions. Their findings provided a weighted average baseline emissions factor of 0.606 tons of CO_2_/MWh for wind and solar power projects in Pakistan, underlining the potential for carbon emissions reductions through renewable-based power projects. A 5 MW SPV power plant was designed using PVsyst software for 50 Iranian cities, with Bushier having the highest capacity factor and Anzali having the lowest, for a mean capacity factor of 22.27%^35^. In Serbia^36^, studies were conducted to estimate the potential for producing electricity using 1 MW solar power plants employing the various types of solar PV modules available, and it was discovered that CdTe solar modules produce more electricity. Elhodeiby et al.^37^ provided a performance analysis of a 3.6 kW rooftop grid-connected solar photovoltaic system in Egypt. All the electricity generated by the system was routed into the 220 V, 50 Hz low voltage grid and monitored for a year. Veerendra Kumar et al.^38^ in 2018 and 2019, the United States (USA) produced 10.6 GW and 13.3 GW of solar photovoltaic (PV) panels, respectively. The cumulative operating photovoltaic capacity in the United States topped 76 GWDC by the end of 2019, up from just 1 GW at the end of 2009. Under the framework of research and development, the International Energy Agency (IEA) Photovoltaic Power Systems Programme (PVPS) has specified a set of 13 aims for the expansion of operation, performance, and monitoring of solar photovoltaic plants. Recently, large-scale photovoltaic power plant studies have been done^39^. References^40,41^ did a study on solar power plants (1523 kW and multi-MW) located in the Canaries (Spain), they discovered that the measured specific yields were within 3% of the simulated findings, these solar power plants were meticulously managed and located in the best possible places. Researchers utilized PVsyst to examine the potential of 44 Saudi Arabian locations for grid-connected solar power plants with a 10 MW installed capacity. The tool assisted them in forecasting energy output, greenhouse gas GHG) emissions, and financial aspects of the proposed solar power plants^42^. According to Satsangi et al.^43^ Indian 40 kWp GIPV system had photovoltaic array efficiency of 9.36%, inverter efficiency of 90.9%, and overall system efficiency of 8.51%. In another study^44^, Antonanzas et al. assessed a 12-kW solar power plant using the International Solar Project Model. They discovered that the best-case scenario for this plant was to meet power demand using 27% solar energy and 73% grid electricity. They were able to achieve a minimum reduction in greenhouse gas emissions of 23 t of CO_2_ per year by using this strategy. A structured methodology was utilized to examine the integration of solar plants into weak distribution systems in grid connection studies^45^. The performance ratios, yield energies, reference energies, capacity utilization factors, and energy efficiency of various solar photovoltaic systems were also examined^46^. Various research projects throughout the world have conducted detailed studies of solar photovoltaic (PV) systems, revealing light on their performance in a variety of climates and situations for instance in Pakistan, operating in a similar semi-arid environment, studies found that TF-Si technology outperformed OPV, CIGS, and TF-Si systems inside a 100 MW installation, particularly in humid conditions^47^. In a 13 MW configuration using bifacial and CIGS technologies, energy generation in South Korea’s semi-arid region was slightly lower than expected^48^. In Iran’s arid desert, a 10 MW installation using Poly-Si, A-Si, and OPV technologies revealed that the highest maximum power output occurred in July, with OPV having a significant advantage^49^. In Sweden, the study investigated hybrid systems integrating CIGS, Mono-Si, and Poly-Si technologies, finding promise for improvement but recognizing the problem of competing with traditional PV modules and flat plate collectors^50^. Meanwhile, in Spain’s sun-drenched expanse, the inquiry spans six power facilities outfitted with a variety of PV technologies and tracking systems, including fixed tilt, single-axis, and dual-axis setups^51^. Singapore, known for its tropical climate, used modeling findings to determine the best placements and inclinations for solar panels, focusing on east-facing façades and panel slopes of 30 and 40 degrees^52^. Maritime Germany’s exploration of power plants, on the other hand, found that panels with 95% bifaciality outperformed those with 50%, boasting an amazing 7% gain in yield^53^. Utility-scale comparison studies of photovoltaic (PV) systems have been done to discover the most efficient module technology^54^. In Kuwait, for example, an 11.15 MW solar PV plant was examined, with two PV technologies pitted against each other: a 5.5 MW thin-film installation and a 5.6 MW polycrystalline silicon installation. This comparison investigation revealed no major differences between the two methods^55^. Individual modules were also subjected to rigorous testing, with the module performance ratio (PR) for 23 different solar module technologies assessed across four different sites over the course of a year^56^. Burssens et al.^57^ conducted a comprehensive assessment of eight PV technologies in Belgium under difficult climate circumstances. Their findings revealed that monocrystalline silicon (mono-Si), polycrystalline silicon (poly-Si), and heterojunction (HIT) modules performed best in high irradiance environments, while CIGS modules performed well in low irradiance areas. Building-integrated photovoltaics, bifacial modules with different orientations, microgrids, car park shelters, and rooftop systems were also investigated, these studies contribute to a better knowledge of PV technology performance in various circumstances, assisting in the development of more efficient solar energy systems^58,59^.

## Emission reduction potentials of photovoltaic power station

Climate change is an obvious truth, and Pakistan is one of the countries hardest hit by its impacts. The country has the formidable task of combating climate change, with climate-related calamities taking countless lives and causing significant economic losses each year^60^. Pakistan’s energy and power industries have long been substantial contributors to Greenhouse Gas emissions, owing to the country’s strong reliance on fossil fuels for electricity generation. The answer to reducing these emissions is to switch to cleaner and renewable energy sources^61^. It should be noted that the economic rewards from such activities frequently do not fully pay the expenditures of emission reduction. Climate finance instruments, such as the Clean Development Mechanism, can help to promote the adoption of renewable energy technologies (RET) that reduce emissions. The overuse of fossil fuels is a major driver of this transformation. Carbon-based fossil fuel combustion not only generates CO_2_ but also a variety of pollutants, including particulate matter, worsening climate change^62^. Notably, the energy sector bears a disproportionate share of the burden, accounting for nearly three-quarters of carbon dioxide emissions, one-fifth of methane emissions, and a significant amount of nitrous oxide. According to World Bank data^63^, Pakistan’s carbon emissions have increased dramatically during the 1980s, with an annual growth rate of roughly 6.4%. While the pace of growth slowed after 2004, it remained above the global average, with emissions totaling 432.50 million tons (Mt) of CO_2_ equivalent in 2019 and 679.34 million tons (Mt) in 2022. Photovoltaic power plants, an important component of renewable energy, have enormous potential for lowering carbon emissions and improving the environment. Their impact on the environment is fairly minor. This study adopts the United Nations Framework Convention on Climate Change (UNFCCC)^64^ technique for computing marginal carbon emissions to analyze the carbon emission reductions resulting from investments in photovoltaic power. Given Pakistan’s high electricity demand, cumulative electricity generation from prioritized photovoltaic power projects over their operational lives might reach a mind-boggling 50.15 billion kWh, enough to power over 250,000 local houses^65^. When the power generation data for each solar power project is combined with the marginal carbon emission factors, the average yearly carbon emission reduction ascribed to these priority projects is predicted to be 28.23 million tons (Mt) of CO_2_-equivalent. This overall carbon emission decrease totals an astonishing 27.45 million tons (Mt) of CO_2_-equivalent throughout the 25-year service span^66^. This illustrates the significant environmental advantages and carbon emission reductions feasible through investments in photovoltaic power installations, underlining their critical role in Pakistan’s fight against climate change.

## Methodology for system parameters of the PV system

The International Energy Agency developed the performance measures to assess the efficiency of grid-connected solar PV installations^67,68^. These characteristics include energy output, solar resource usage, and total system losses, allowing for a full assessment of the system’s performance. The performance ratio, final PV system yield, and reference yield are among the essential criteria examined. These indicators add up to a full assessment of the solar PV system’s ability to harness solar energy and its overall efficiency in turning sunshine into electricity. Within the scope of our analysis, the most relevant parameters include energy output, array yield, final yield, reference yield, module efficiency, inverter efficiency, system efficiency, energy loss (comprising array capture loss and system loss), performance ratio, and capacity factor. These standardized indicators, recognized as essential benchmarks, form the basis for evaluating the performance of our large-scale photovoltaic power plant allowing for an effective and comparative assessment of its efficiency and productivity. The “Array Yield” is the time it takes the PV plant to produce array DC energy (E_a_) at its nominal solar generator power (P_o_). The values are kilowatt-hours per day per kilowatt peak (kWp). This statistic is used to assess the efficiency and productivity of a PV plant, taking into account variations in solar resource availability and system performance (1), (2)^69^,

1 $${{\text{Y}}_{\text{A}}}=\,{{\text{E}}_{\text{a}}}/{{\text{P}}_{\text{o}}}{\text{h}}$$

2 $${{\text{E}}_{\text{a}}}={\text{ IDC }}*{\text{ VDC }}*{\text{ t }}\left( {{\text{kWh}}} \right)$$

The “Reference Yield” is determined by dividing the total in-plane irradiance by the reference irradiance. It quantifies the peak sun hours or insolation in units of kWh/m^2^ and characterizes the solar radiation resource accessible to the system (3), (4)^69^. It represents the obtainable energy under ideal conditions. The reference yield describes the solar radiation resource for the PV. system and is affected by the PV array’s position, orientation, and month-to-month and year-to-year weather fluctuation. The reference yield is influenced by factors such as the geographical location and orientation of the photovoltaic array, as well as the variability in weather conditions both on a monthly and annual basis^70,71^,

3 $${{\text{Y}}_{\text{R}}}=\,\left[ {{\text{kW h}}/{{\text{m}}^{\text{2}}}} \right]/\,\left[ {{\text{1 kW}}/{{\text{m}}^{\text{2}}}} \right]$$

4 $${{\text{Y}}_{\text{R}}}=\,{{\text{H}}_{\text{t}}}/{{\text{G}}_{\text{o}}}$$

where, H_t_ = Total Horizontal irradiance on array plane (W h/m_2_) and G_o_ = Global irradiance at STC (W/m^2^).

The term ‘Final Yield’ (YF) refers to the total alternating current (AC) energy produced by the PV system over a specified time period. It represents the number of hours the PV array would have to run at maximum capacity to generate the same quantity of electricity. This parameter is crucial in determining the efficiency and performance of a solar PV system (5)^72^,

5 $${\text{YF}}\,=\,{\text{EPV}},{\text{ AC}}/{{\text{P}}_{{\text{max G}},{\text{ STC}}}}$$

The capacity factor is an important parameter in assessing the overall performance and productivity of a power generation system, showing its ability to produce energy consistently through time. The inverter efficiency, also known as conversion efficiency, is determined by the ratio of AC power generated by the inverter to DC power produced by the PV array system, and the system efficiency is calculated as PV module efficiency multiplied by inverter efficiency, (6)–(9)^72^,

6 $$\:\text{C}\text{F}\:=\:\text{a}\text{c}\text{t}\text{u}\text{a}\text{l}\:\text{o}\text{u}\text{t}\text{p}\text{u}\text{t}\:\text{A}\text{C}\:\text{e}\text{n}\text{e}\text{r}\text{g}\text{y}/\text{r}\text{a}\text{t}\text{e}\text{d}\:\text{a}\text{r}\text{r}\text{a}\text{y}\:\text{c}\text{a}\text{p}\text{a}\text{c}\text{i}\text{t}\text{y}=\:\text{E}\text{A}\text{C}/\:(\text{P}\text{n}\text{o}\text{m}\:\times\:\:24)\times\:\:100\:\left(\text{h}\text{o}\text{u}\text{r}\text{s}\right)$$

7 $$\:{\text{P}\text{h}\text{o}\text{t}\text{o}\text{v}\text{o}\text{l}\text{t}\text{a}\text{i}\text{c}\:\text{E}\text{f}\text{f}\text{i}\text{c}\text{i}\text{e}\text{n}\text{c}\text{y},\:\tau\:}_{PV}=\:\text{E}\text{D}\text{C}/\:(\text{P}\text{V}\text{a}\text{r}\text{e}\text{a}\:\times\:\:\text{i}\text{r}\text{r}\text{a}\text{d}\text{i}\text{a}\text{t}\text{i}\text{o}\text{n})$$

8 $$\:{\text{I}\text{n}\text{v}\text{e}\text{r}\text{t}\text{e}\text{r}\:\text{E}\text{f}\text{f}\text{i}\text{c}\text{i}\text{e}\text{n}\text{c}\text{y},\:\tau\:}_{In}=\:(\text{i}\text{n}\text{v}\text{e}\text{r}\text{t}\text{e}\text{r}\:\text{e}\text{x}\text{p}\text{o}\text{r}\text{t}/\text{E}\text{D}\text{C})$$

9 $$\:{\text{S}\text{y}\text{s}\text{t}\text{e}\text{m}\:\text{E}\text{f}\text{f}\text{i}\text{c}\text{i}\text{e}\text{n}\text{c}\text{y},\:\tau\:}_{s}=\:\text{E}\text{A}\text{C}\:/\:(\text{P}\text{V}\text{a}\text{r}\text{e}\text{a}\:\times\:\:\text{i}\text{r}\text{r}\text{a}\text{d}\text{i}\text{a}\text{t}\text{i}\text{o}\text{n})$$

Natural energy losses occur in different components of a grid-connected Solar Photovoltaic (SPV) power plant operating under real-world conditions. The monitored data generated from the system’s performance is used to analyze these inherent losses. Energy losses in solar photovoltaic (SPV) power plants are unavoidable due to a variety of variables. Understanding and correcting these inefficiencies is critical for improving plant efficiency and energy generation. Monitoring effectively delivers vital insights for improved performance (10)^72^,

10 $${{\text{L}}_{{\text{CM}}}}=\,{{\text{Y}}_{{\text{CR}}}} - {{\text{Y}}_{\text{A}}}$$

These losses are attributed to cell temperatures surpassing 25 °C, leading to thermal inefficiencies. The thermal capture loss (LCT) is quantified as the difference between the reference field and the corrected reference field (11), (12)^72^,

11 $${{\text{L}}_{\text{C}}}=\,{{\text{Y}}_{\text{R}}} - {{\text{Y}}_{\text{A}}}$$

12 $${{\text{L}}_{\text{S}}}=\,{{\text{Y}}_{\text{A}}} - {{\text{Y}}_{\text{F}}}$$

Losses generated by energy conduction within the photovoltaic modules contribute to miscellaneous capture loss (LCM). Addressing these diverse factors plays a crucial role in minimizing losses and optimizing the SPV power plant’s overall performance.

To estimate the carbon footprint reduction achieved by the solar power plant, we used PVsyst software to simulate the system’s performance and calculate the avoided CO_2_ emissions. The software uses the following equations to compute the CO_2_ reduction (13)-(15)^73,74^,

13 $${\text{AEP}}\,=\,{\text{Installed capacity x specific yield}}$$

14 $${\text{C}}{{\text{O}}_{\text{2}}}{\text{Emission reduction}}\,=\,{\text{AEP x emission factor}}$$

15 $${\text{Total C}}{{\text{O}}_{\text{2}}}{\text{savings}}\,=\,{\text{C}}{{\text{O}}_{\text{2}}}{\text{Emission reduction x plant lifetime}}$$

Where AEP represents the annual energy production in kWh/year, the installed capacity is in kWp, and the specific yield is in kWh/kWp/year. The CO_2_ emission reduction is measured in tons per year, the emission factor is in kg CO_2_ per kWh, the total CO_2_ savings are in tons, and the plant lifetime is measured in years.

The carbon footprint reduction per installed kWp is measured in tons of CO_2_ per kWp, and the annual CO_2_ reduction per kWp is measured in tons/kWp/year (16) and (17)^73,74^,

16 $$\:CO2\:reduction\:per\:kWp=\frac{Total\:CO2\:savings}{Installed\:capacity}$$

17 $$\:Annual\:CO2\:reduction\:per\:kWp=\frac{CO2\:emission\:reduction}{Installed\:capacity}$$

## Selection criteria and proposed system

### PV panel

**Fig. 3:**
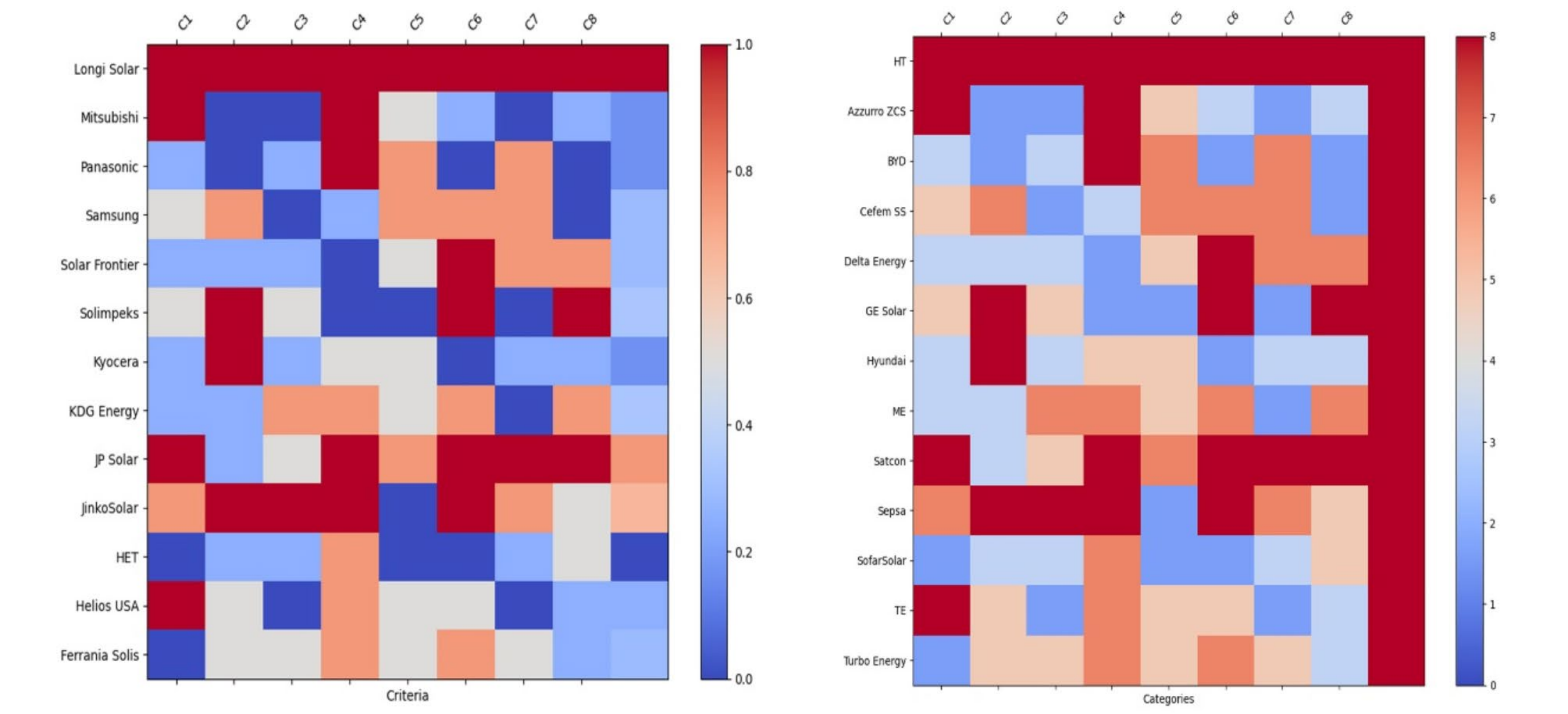
Decision matrix for the selection of (**a**) solar PV, (**b**) inverter.

We started with 13 possibilities in our search for the best PV panels for our solar plant (Longi Solar, Mitsubishi, Panasonic, Samsung, Solar Frontier, Solimpeks, Kyocera, KDG Energy, JP Solar, JinkoSolar, Hulk Energy Technology (HET), Helios USA, Ferrania Solis). These options were thoroughly evaluated based on the following major criteria (efficiency, cost, warranty, compatibility, monitoring, cost-effectiveness, safety, and environmental impact) mentioned in Fig. 3a as C1, C2, C3…., C8. Our data-driven methodology gave each criterion an equal weight of 8 points. Longi Solar emerged as the best option after this evaluation. While Solimpeks and Kyocera were less expensive, they had restrictions. Mitsubishi Electric excelled in capacity but had to make sacrifices. Solar Frontier outperformed in terms of environmental performance but suffered efficiency and compatibility issues. This thorough methodology offers a data-backed selection customized to your long-term energy objectives.

### Inverter

We started with 17 possibilities in our search for the best inverter for our solar plant. These inverters, which included respectable names like Huawei Technologies, Azzurro ZCS, BYD, Cefem Solar System, Delta Energy, GE Solar, Hyundai, Mitsubishi Electric, Satcon, Sepsa, SofarSolar, Tabuchi Electric, and Turbo Energy, were meticulously scrutinized across a matrix of critical criteria. These criteria included Inverter Efficiency, Capacity and Sizing, Reliability and Warranty, Compatibility, Monitoring and Communication, Cost and Budget, Safety and Grid Compliance, and Environmental Impact mentioned in the Fig. 3 (b) as C1, C2, C3…., C8. Using an equal weight of 8 points for each criterion, our data-driven review highlighted the advantages and disadvantages of each inverter option. While Huawei Technologies emerged as the clear winner, with superior performance in all areas, it’s important to note that alternatives such as Sepsa, SofarSolar, Tabuchi Electric, and Turbo Energy held their own, demonstrating unique capabilities that may correspond with specific project requirements. This rigorous procedure ensures that your selection aligns with your solar energy requirements and environmental goals.

### Site selection

**Fig. 4:**
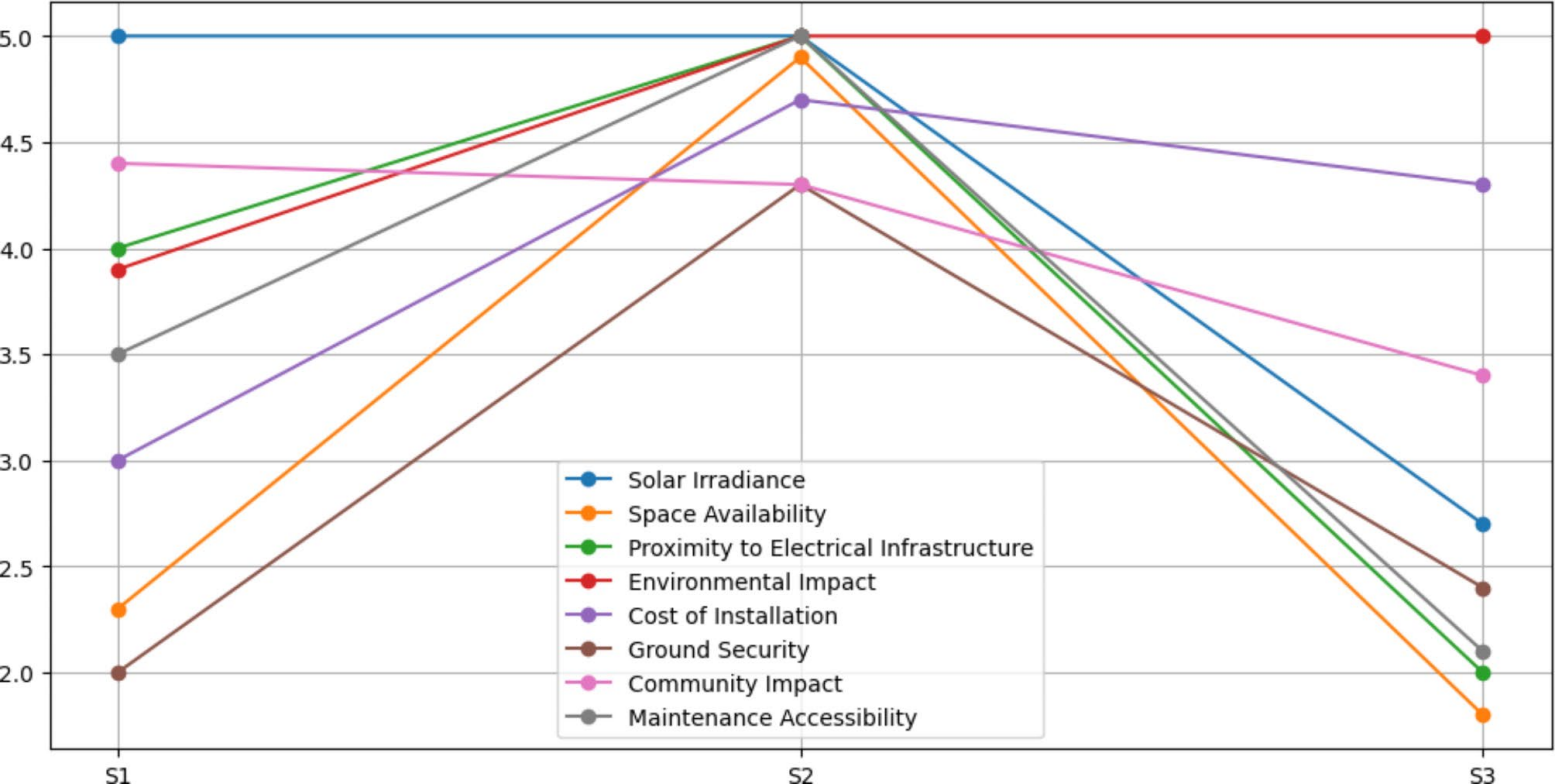
Comparison of criteria for given 3 sites for the SPV at NUST.

We conducted a thorough analysis utilizing a decision matrix that included different crucial factors required for solar plant installation to pick the optimal site. The evaluation procedure entailed examining three separate sites, namely S1, S2, and S3, based on important characteristics that greatly determine each location’s potential. Solar Irradiance, Space Availability, Proximity to Electrical Infrastructure, Environmental Impact, Cost of Installation, Ground Security, Community Impact, and Maintenance Accessibility were among the criteria considered. The combined line graph (Fig. 4) provide a clear and instructive visual picture of how each site performed in relation to these critical criteria. Site S2 consistently outperformed, receiving the top marks across the bulk of the tested categories. Notably, it outperformed in Solar Irradiance, Space Availability, Proximity to Electrical Infrastructure, and Environmental Impact. Because of this aggregate strength, Site S2 is a particularly advantageous location for solar plant development, providing excellent conditions for efficient energy generation. Site S1, while not outperforming S2, performed admirably in numerous areas, particularly Solar Irradiance, Proximity to Electrical Infrastructure, and Community Impact. Site S3, on the other hand, while showing potential in terms of Solar Irradiance and Community Impact, confronts problems in terms of Space Availability, Installation Cost, and Environmental Impact. In conclusion, this comprehensive analysis and the resulting graphical representation enable decision-makers to make well-informed choices when selecting the most suitable site for solar plant installation, Site S2 emerges as the preferred choice, presenting a harmonious balance of favorable criteria that aligns with the project’s objectives and environmental considerations.

## Overview of the grid-connected solar PV system at NSTP-NUST

**Fig. 5:**
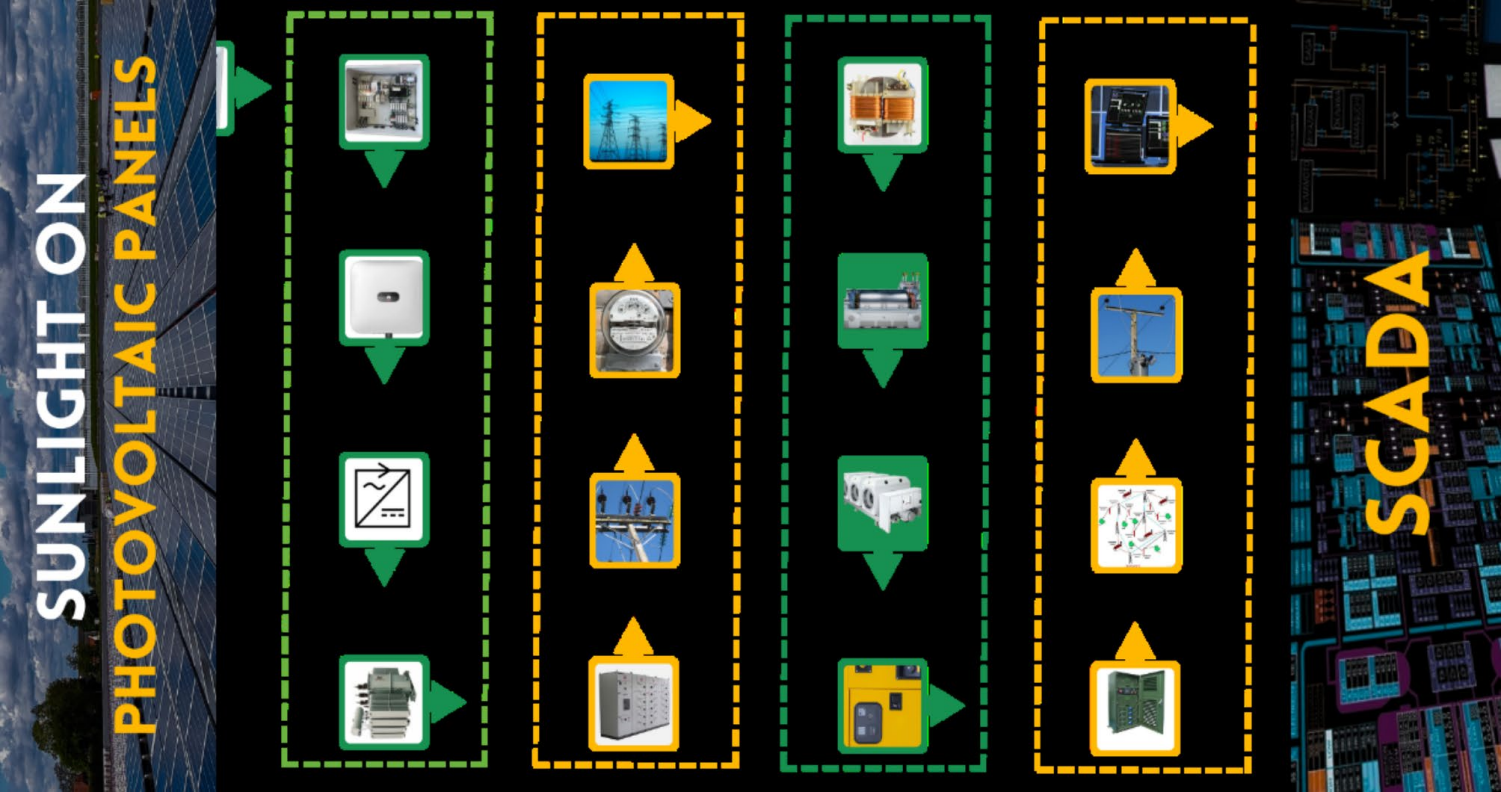
Layout of 8.79 MW gid-connected solar power plant at NUST.

The schematic diagram in Fig. 5 outlines the layout of an advanced 8.79 MW solar power facility at the National Sciences and Technology Park (NSTP) within the National University of Sciences and Technology (NUST) in Islamabad, Pakistan. PV modules efficiently gather solar energy and convert it from direct current (DC) to alternating current (AC) power utilizing modern inverters and transformers. High-tension switchgear guarantees that energy is transferred seamlessly to the 33 kV input/output (I/O) switchyard, where it is metered before being integrated into the utility grid. The lower half focuses on the generator-grid link, which is regulated by a control system that includes power conditioning for quality assurance. This design maximizes energy generating efficiency while avoiding the need for substantial storage. The geographical location of small, isolated power systems has a considerable impact on performance, with local climate influencing module temperature and energy generation. Precise modeling, including regression analysis, allows for production forecasting, grid integration, and system health prediction, all of which improve overall sustainability.

### Site overview

**Fig. 6:**
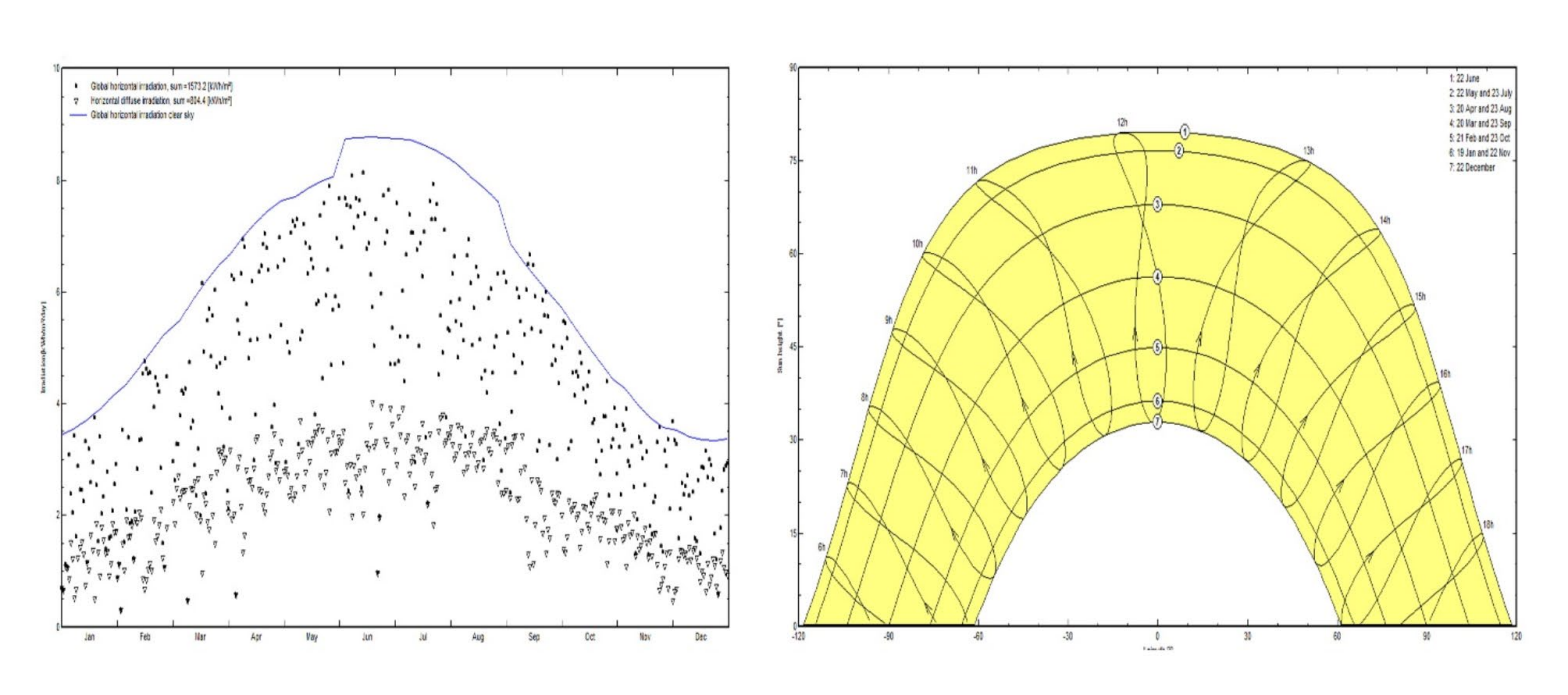
(**a**) Global horizontal, and horizontal diffuse irradiation. (**b**) Sun path and solar angles.

**Fig. 7:**
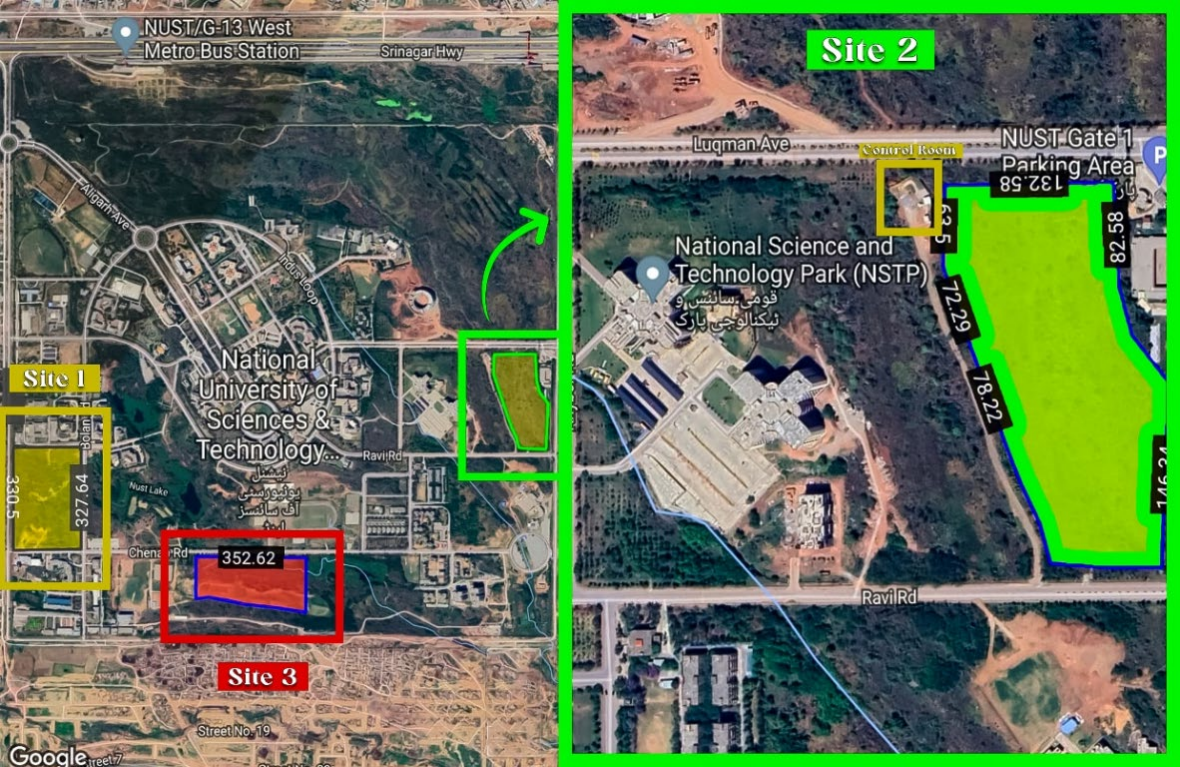
Aerial view of the proposed site location for the SPV (Source: Google Maps, 2024. Used under principles of fair use for educational and non-commercial purposes. Available from: https://www.google.com/maps).

The National University of Sciences and Technology (NUST) in Islamabad, which is located at 33.647279° latitude and 72.99987° longitude, provides a strategic advantage due to its copious year-round sun radiation. Islamabad has consistently high insolation levels, with approximately 2945 h of annual sunshine, which equates to over 6400 trillion kWh of solar energy potential. The detailed yearly climate data is illustrated in Table 1. Furthermore, the region’s high temperatures, which can reach 45.5 °C, contribute to its aptitude for solar power generation. For solar panels in Pakistan, the ideal direction is generally south facing, which corresponds to an azimuth angle of approximately 180°. There is no land rent since the site location is the property and falls under the premises of the National University of Sciences and Technology (NUST). Global horizontal radiation, horizontal diffuse irradiation and global horizontal irradiation, clear sky is shown in Fig. 6(a), while the sun path and solar azimuths in Fig. 6(b). The proposed plant is located at the Northeast of the National Sciences and Technology Park (NSTP) (Fig. 7) and the map data of the selected site is shown in Table 2.

**Table 1:** Monthly climate data for NUST, Islamabad.

Month	Average high temperature (°C)	Average low temperature (°C)	Mean monthly sunshine (h)
January	17.7	2.6	195.7
February	19.1	5.1	187.1
March	23.9	9.9	202.3
April	30.1	15.0	252.4
May	35.3	19.7	311.9
June	38.7	23.7	300.1
July	35.0	24.3	264.4
August	33.4	23.5	250.7
September	33.5	20.6	262.2
October	30.9	13.9	275.5
November	25.4	7.5	247.9
December	19.7	3.4	195.6
Year	28.5	14.1	245.4

**Table 2:** Map data for selected site at the National University of Sciences and Technology (NUST), Islamabad

Parameters	Abbreviations	Values
Total land area for PV plant	L	9.36 acres
Longitude and latitude	LL	33.647279°, 72.99987°
Global horizontal irradiation	GHI	1760.4 kWh/m^2^
Direct normal irradiation	DNI	1493.1 kWh/m^2^
Diffuse horizontal irradiation	DHI	749.9 kWh/m^2^
Global tilted irradiation at optimum angle	GTI_opt_	1968.4 kWh/m^2^
Optimum tilt of PV modules	OPT	30°
Air temperature	TEMP	23.21 °C
Terrain elevation	ELE	552 m
Time zone	UTC	+ 5

### Plant layout

The 11.5 MW solar power facility at NUST, Islamabad, covers 9.36 acres of land and is divided into six strategic blocks, which are further subdivided into twelve sub-blocks totaling 8.79 MW capacity. The system efficiently integrates AC power into the 33 kV grid via a double fed primary winding transformer with 96 × 8 strings in Block A and 40 × 9 strings in Block B, which are coupled to inverters via string combiner boxes. This plant is extremely adaptable, collecting power from the grid during low solar radiation periods to ensure uninterrupted power supply. Its substantial battery storage improves energy stability, cementing its position as a dependable and versatile energy source.

### Tilt selection

**Fig. 8:**
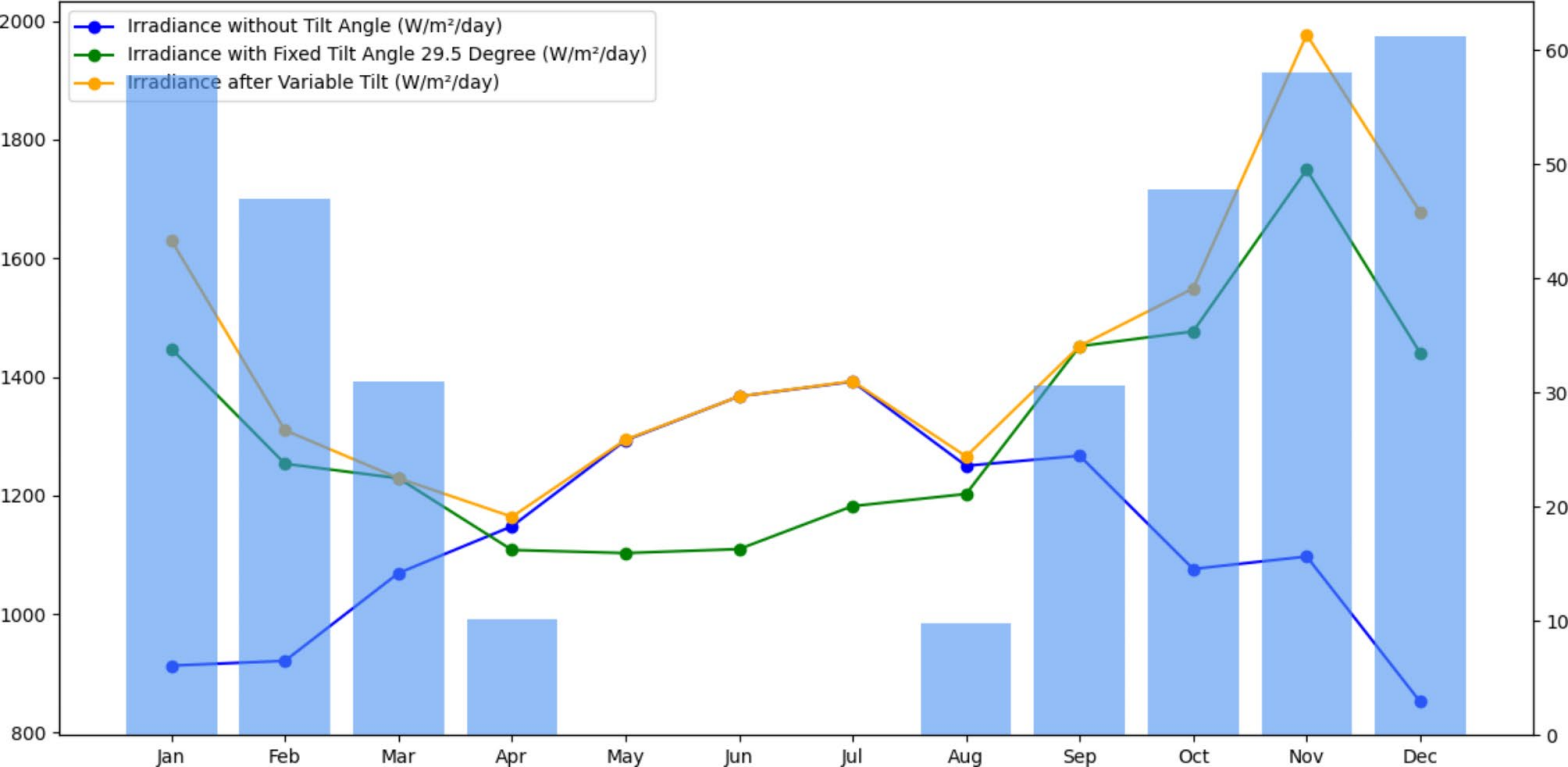
Comparative analysis of solar irradiance levels and tilt angle adjustments over months.

The tilt angle of the PV array is kept equal to the latitude of the corresponding location to get maximum solar radiation^75^. Figure 8 provides an interesting analysis of tilt angle selection and its impact on solar irradiance levels throughout the year in the context of our study on solar energy optimization. This image is made up of numerous crucial aspects, each of which helps us grasp this critical aspect of solar energy generation. The blue line depicts the baseline irradiance levels without any tilt angle modifications. This line graphically depicts the natural change in solar exposure that occurs throughout the year. The green line then shows the impact of using a fixed tilt angle of 29.5°. This consistent tilt angle modifies the solar irradiance pattern, resulting in a consistent but altered level of solar energy throughout the year. Finally, the orange line emphasizes the system’s adaptability through varied tilt changes, it shows how the sun irradiance levels adapt to shifting angles, providing important insights into the system’s adaptability. This chart shows the variable tilt angles in degrees, and it serves as a visual reference for the modifications we performed in our analysis. The combined depiction in Fig. 8 is a valuable tool for determining the sort of tilt system to be utilized, whether it is fixed or seasonal tilt, and the best tilt angle to optimize solar energy output across all seasons. Furthermore, our research found that using seasonal tilt angles resulted in a 36.31% increase in solar irradiation when compared to no tilt scenarios. Furthermore, the average percentage change in irradiance between seasonal tilt and fixed tilt was discovered to be 16.59%, highlighting the advantages of an adaptive tilt strategy for increased solar energy efficiency.

### Electricity consumption and load calculation

**Fig. 9:**
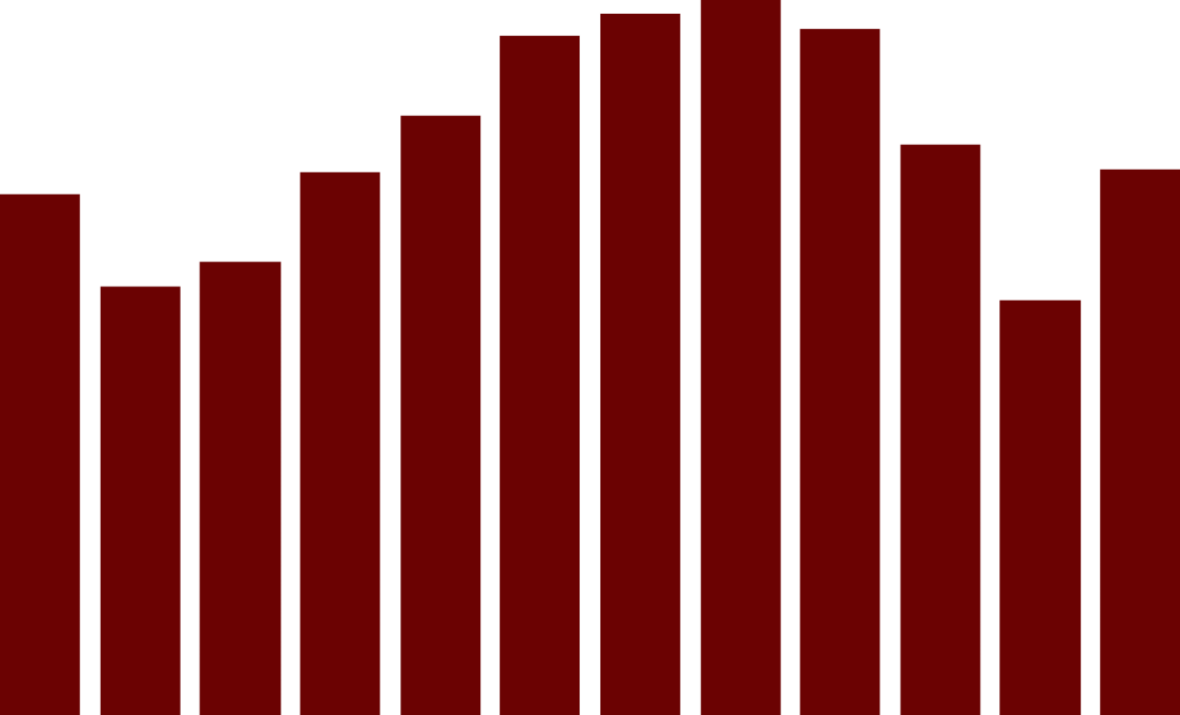
Monthly electricity consumption at the National Sciences and Technology Park (NSTP), NUST in MWh.

The monthly electrical consumption data for the National Sciences and Technology Park (NSTP) at the National University of Sciences and Technology (NUST) solar power plant in Islamabad, Pakistan, shows significant seasonal changes. These swings in consumption are directly related to seasonal and environmental change (Fig. 9). Lower demand is seen during the winter months, gradually increasing when spring arrives, and peaking during the hot summer months. As the year passes and temperatures fall, so does the power demand, which reaches its lowest point in the fall and early winter. These fluctuations in monthly electrical consumption are critical in optimizing the performance of the solar power plant and providing a continuous energy supply in response to seasonal variations.

### Solar PV specifications

**Fig. 10:**
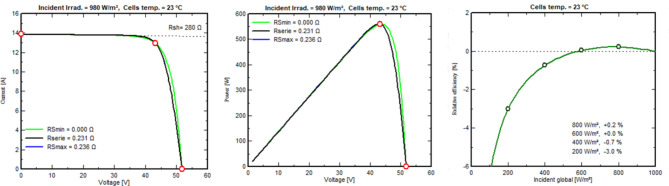
(**a**) I/V curve. (**b**) P/V curve, and (**c**) relative efficiency.

**Fig. 11:**
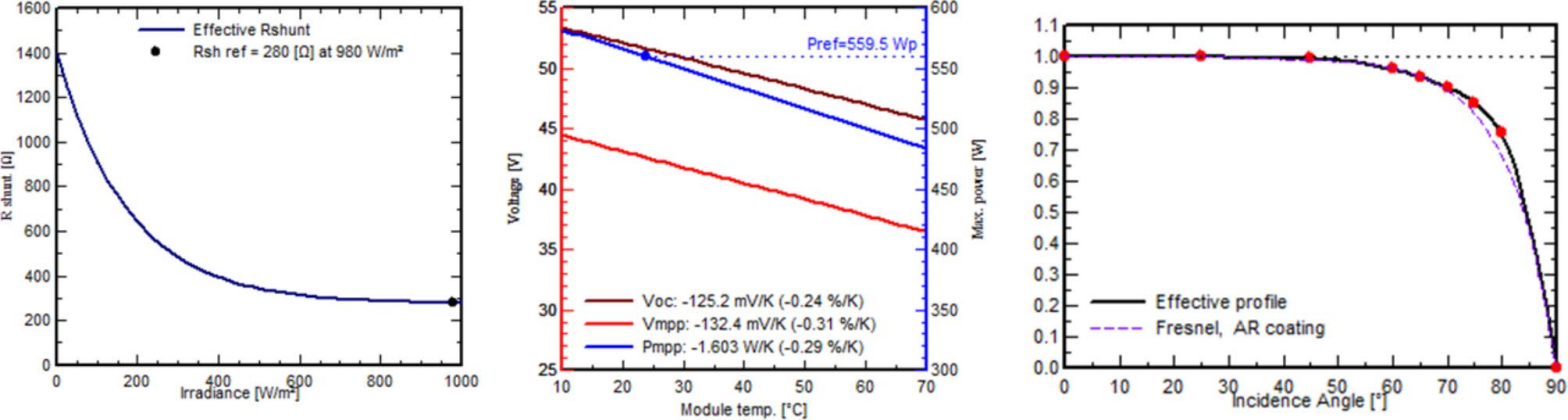
(**a**) Rshunt exponential as irradiance. (**b**) Voltage and power with module temperature, and (**c**) Incidence angle modifier.

**Fig. 12:**
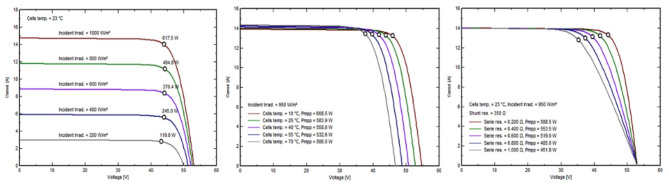
Current vs. voltage behavior of (**a**) Incident irradiation. (**b**) Cell temperature, and (**c**) Series resistance and Pmpp.

**Fig. 13:**
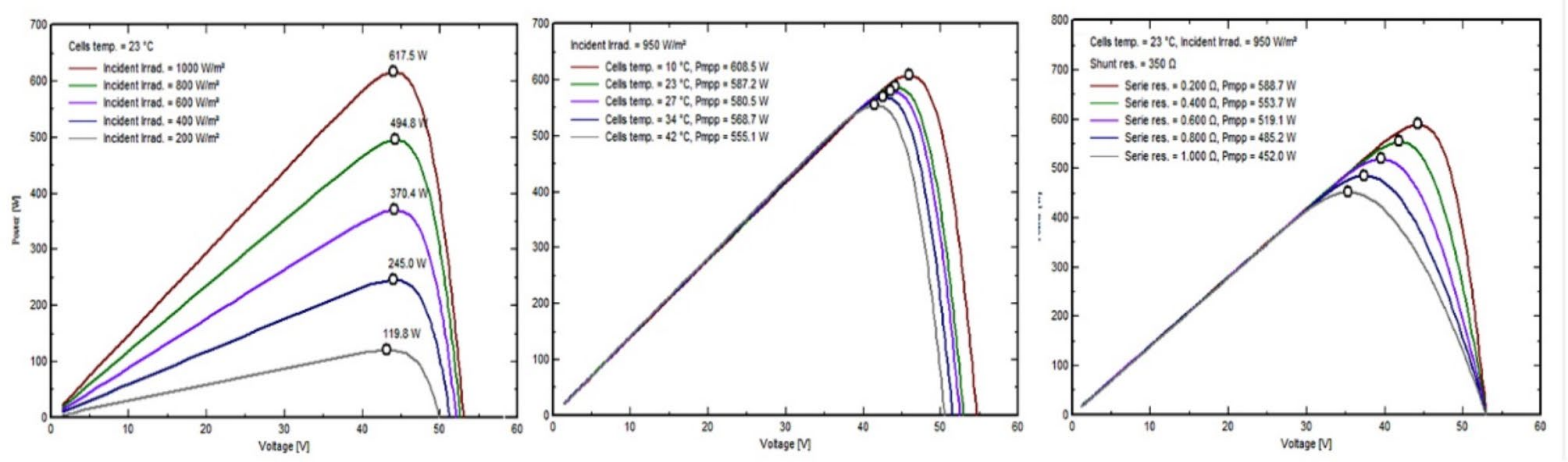
Power vs. voltage behavior of (**a**) Incident irradiation. (**b**) Cell temperature, and (**c**) Series resistance and Pmpp.

**Fig. 14:**
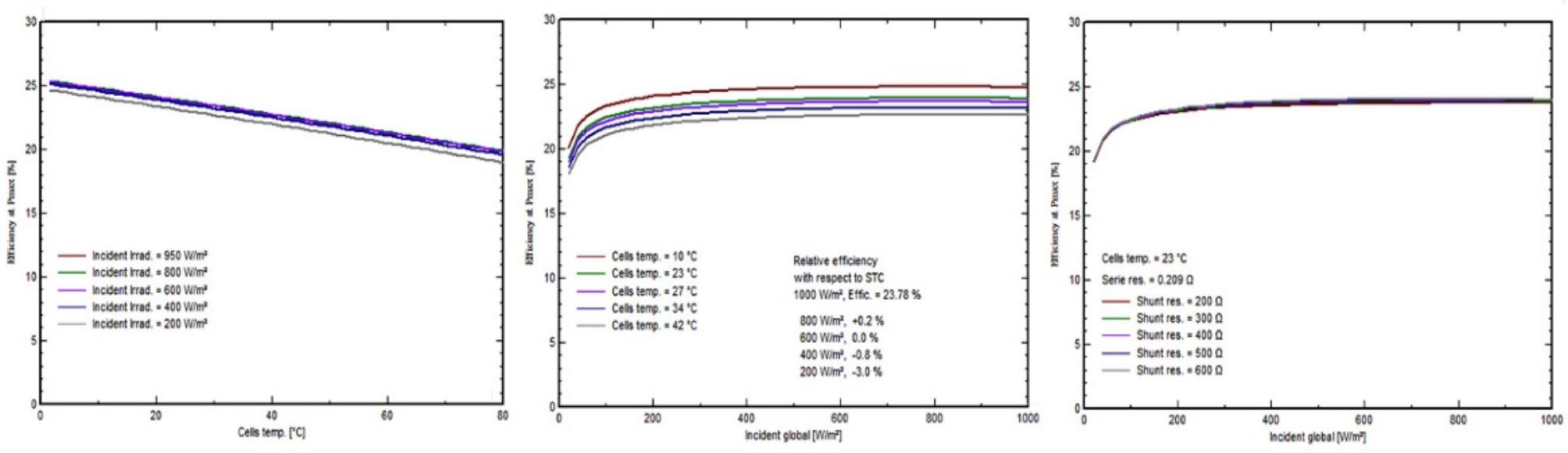
Efficiency at power behavior of (**a**) incident irradiation and cell temperature, (**b**) cell temperature and incident global irradiation, and (**c**). shunt resistance and incident global irradiation.

The solar panels used in this system are Longi_LR5-72_HTH_600M by Longi Solar 14,664 modules, boasting 600 Wp power output detailed specifications in (Table 3) measured at the standard testing conditions which are irradiance of 1000/m^2^, air mass 1.5 g, and cell temperature of 25 °C. These monocrystalline silicon panels were chosen because they are more efficient than other cell types, such as polycrystalline and amorphous silicon. Aluminum frame, Tempered AR glass structures with glass foil, Jbox IP 68, MC4 EVO2 or mateable HPBC Half-Cut connections. With a 3.5-meter structure-to-structure and leg center-to-center distance, as well as small 35-mm gaps between neighboring panels, the work stresses precision. The ground and the bottom module edge are separated by 450 mm. Regular bi-monthly cleaning and high-density encapsulation technologies improve performance by 0.019–0.230%. Figure 10 depicts the critical model parameters for our investigation. In (a), (b), and (c), we detail the shunt resistance (Rsh) at 280 ohms, the series resistance model (Rs) at 0.231 ohms (with a maximum of 0.235 ohms), the apparent dV/dI at 0.37 ohms, the diode saturation current (IoRef) at 0.019 nA, and the diode quality factor (Gamma) at 1.003. In Fig. 11, (a) shows the exponential behavior of Rsh as a function of incident irradiance, with Rshunt set to 1400 ohms and an exponential value of 5.5. (b) shows the temperature coefficients muPMax and muVoc, emphasizing their gamma dependence and serving as critical output parameters for the classic one-diode model, and (c) shows the incidence angle modifier’s behavior on the effective profile. Similarly, Fig. 12 shows the current vs. voltage behavior of (a) Incident irradiation (b) cell temperature, and (c) series resistance and P_mpp_. Figure 13 shows power vs. voltage behavior of (a) incident irradiation (b) cell temperature, and (c) series resistance and P_mpp_. Figure 14 illustrates the efficiency at power behavior of (a) incident irradiation and cell temperature (b) cell temperature and incident global irradiation, and (c) shunt resistance and incident global irradiation. These factors are essential for comprehending our system’s behavior. This all-encompassing approach provides maximum energy generation and system efficiency.

**Table 3:** Selected PV module specifications.

Parameters	Abbreviations	Values
Peak power	P_max_	600 Wp
Rated Voltage	V_rd_	33.647279°, 72.99987°
Tolerance	Tol	± 3.0%
Rated current	I_rd_	1760.4 kWh/m^2^
Short circuit current	I_SC_	14.460 A
Open circuit voltage	V_oc_	52.81 V
Maximum current point	I_mpp_	13.440 A
Maximum voltage point	V_mmp_	44.66 V
Temperature coefficient	muI_SC_	7.2 mA/^o^C
Efficiency/cell atea	Eff	25.75%
Maximum power	TEMP	23.21 ^o^C
Size	L × W × T	2278 × 1134 × 35 mm
Module weight	W	27.50 kg
Module area	A	2.583 m^2^

### Inventor specifications

**Fig. 15:**
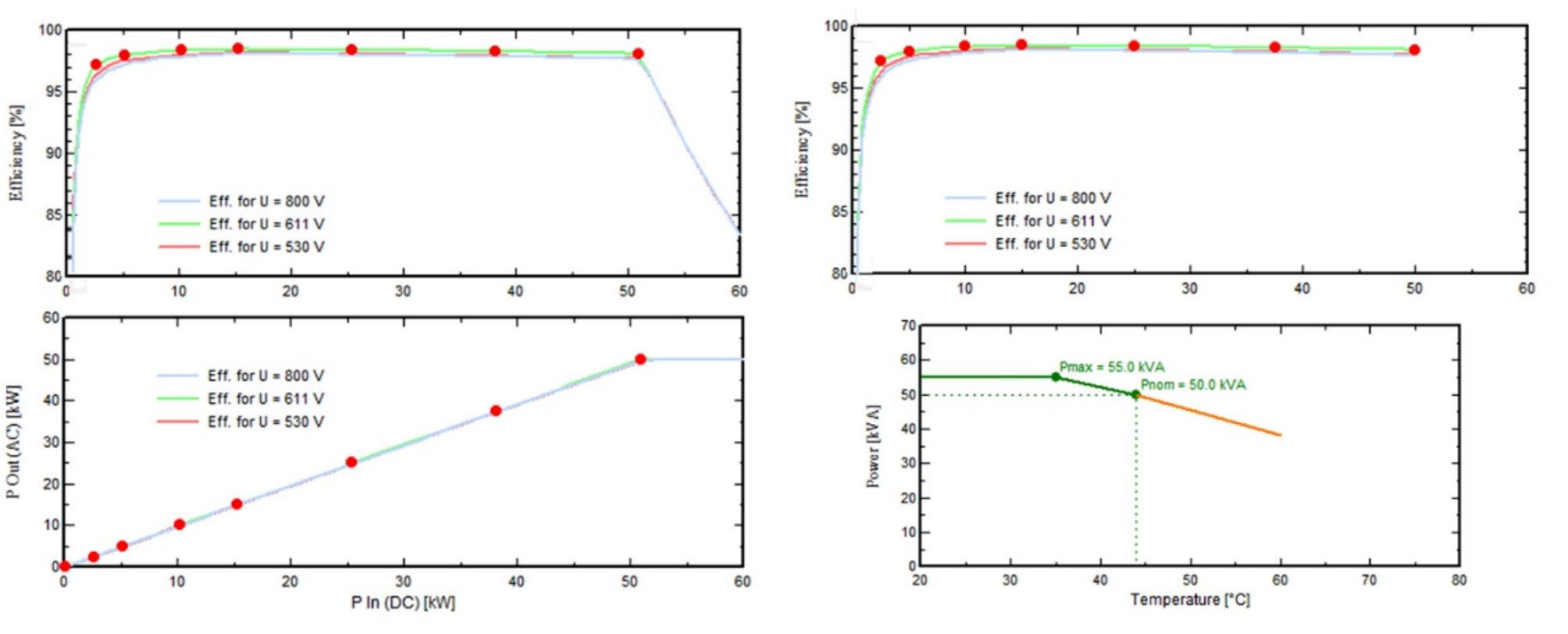
Power in DC comparison with (**a**) Top left—efficiency power in (**b**) top right—efficiency power out (**c**) bottom left—power out in AC, and (**d**) bottom right—maximum power with temperature.

In this grid-tied solar photovoltaic (PV) system, the inverters play a crucial role in converting DC power into AC power. The Huawei Technologies SUN2000-50KTL-M3-380 V, H inverter was chosen. It has a 380 V operating voltage and a dual stage, detailed specifications in Table 4 measured at the standard testing conditions which are irradiance of 1000/m^2^, air mass of 1.5 g, and cell temperature of 25 °C. LED indicators, integrated WLAN compatibility, and the FusionSolar APP for monitoring are among the control features. This inverter works with SUN2000 1100/1300 W optimizers. The system in Fig. 15 operates at a medium voltage of 611 V and has an amazing inverter efficiency of 98.26%. The configuration is intended for an average temperature of 23.1 °C. It has a high-power factor (cos) of 1.00 and tan values that ensure optimal performance. At about 35 °C, the inverter handles an apparent power of KVA and generates a nominal AC power of 50 KVA, with a maximum AC power output of 55 KVA. For safety reasons, the power limiter is set at 45.5 kWac at 50 °C and 38.2 kWac at 60 °C. Four MPPT inputs, 5.5 W night consumption, I_sol_ monitoring, a DC switch, and adjustable ENS and AC disconnect options are also included. This all-encompassing approach provides maximum energy generation and system efficiency for this solar plant.

**Table 4:** Selected inverter specifications

Parameters	Abbreviations	Values
Minimum MPP voltage	Mpp	200 V
Nominal MPP voltage	Npp	600 V
Maximum MPP voltage	Mpp_max_	1000 V
Absolute max PV voltage	Mpp_ab_	1100 V
Power threshold	P_tre_	75 W
Grid voltage	G_v_	380 V
Output side	Category	Tri phased
Size	W × D × H	640 × 270 × 530 mm
Weight	Weight	48 g

### Power evacuation

This 8.78 MW solar power plant’s transformer is rated at 1.5 MVA and has the Vector group designation DY5Y5 four-winding transformer (double story transformer). It has a 390 V primary voltage and is directly connected to the 33 kV switchyard on the secondary side. The primary current rating is 2.24 A, and the secondary current rating is 980 A, proving its capacity to handle power needs efficiently. The transformer has an excellent efficiency of around 96.45%, which ensures low energy losses during conversion. It works at a nominal power of 9.65 MVA under standard test conditions (STC), with an iron loss of 1158 kVA, or around 12% of the nominal power. The copper loss is 113.33 kVA, which is equal to 100% of the nominal power, and the coils have an equivalent resistance of 3 × 0.27 m s.

### Data monitoring

A weather monitoring station situated adjacent to the power plant in the (Southwestern Edge) records data on wind speed, ambient temperature, and solar radiation (Fig. 9). The control room of the solar power plant is outfitted with cutting-edge equipment for real-time monitoring and optimization. A SCADA system provides precise control, while data analytics software analyzes sensor data for trend analysis and maintenance forecasting. Data transfer is ensured by communication infrastructure, and the facility is protected by security mechanisms. An Energy Management System (EMS) manages energy distribution, while predictive maintenance technologies maintain component health and an easy-to-use Human-Machine Interface (HMI) assists operators. Backup power systems ensure continuous operation, allowing for efficient and dependable energy generation.

## Simulations using PV SYST

The amount of solar energy received per unit area, known as solar irradiance, has a considerable impact on the peak power production of solar modules. Higher irradiance levels result in enhanced power generation, while lower levels result in decreased output. The link between temperature and power output, on the other hand, is more complicated. Even with enough solar exposure, rising temperatures cause a decrease in power output above a particular threshold. This temperature-related effect must be taken into account for improving solar system performance. Furthermore, even with constant solar irradiation, power generation drops significantly as the ambient temperature rises due to thermal characteristics. Understanding the relationship between sun irradiance, temperature, and power production is critical for increasing energy efficiency in the design and operation of solar power systems.

### Balances and main results

**Fig. 16:**
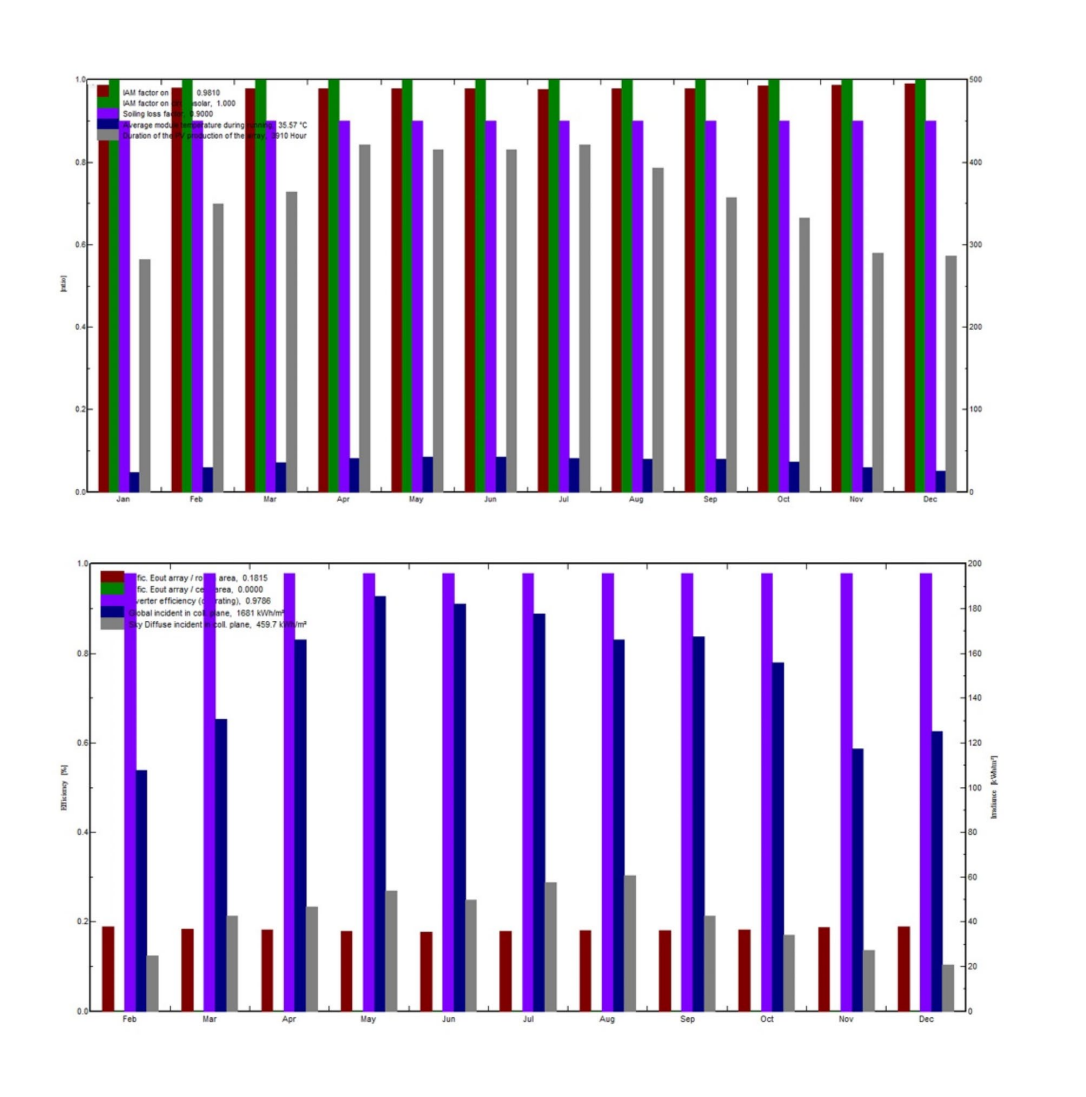
Behavior of major performance parameters of solar power plant.

**Fig. 17:**
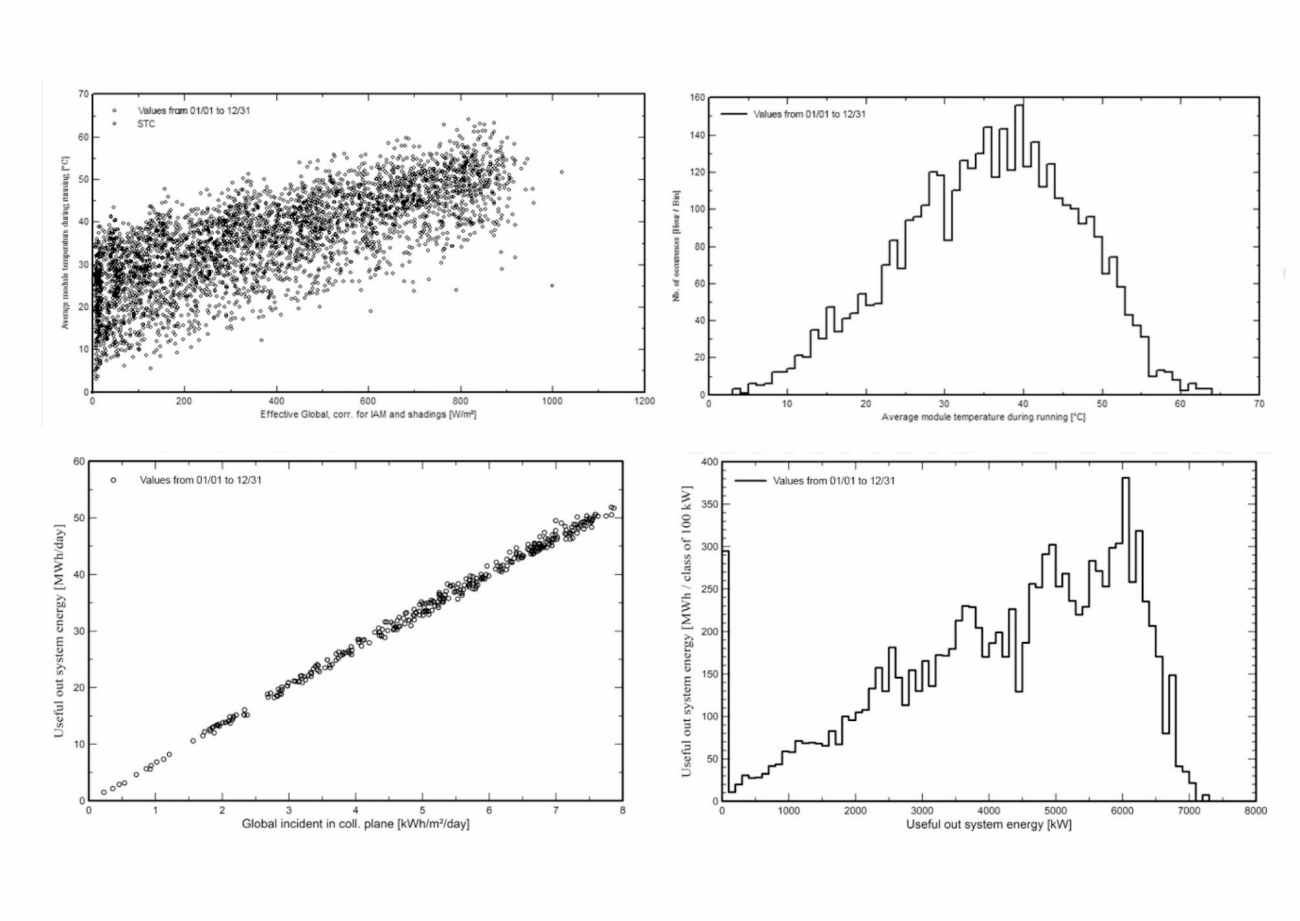
Behavior of average module temperature during running with (**a**) Top left—effective global correction factor (**b**) top right—number of occurrences (**c**) bottom left—Useful system energy vs. global incident energy, and (**d**) bottom right—percentage of useful system energy.

The annual global horizontal irradiation is 802.04 kWh/m^2^, and the global incident energy incident on the collector plane annually is 1580 kWh/m^2^. The total energy obtained from the output of the PV array is 12,343,298 kWh, the effective grid value comes out to be 11,539,692 kWh, the effective solar value is 559,187 kWh and the effective energy to the grid is 6,715 kWh (Table 5). The annual efficiency (E_out_) of the PV array concerning the rough area is 21.42%. Figure 16 depicts a thorough overview of major solar PV system performance parameters. The particular E_out_ array for the rough region is estimated at 0.1815, stressing its potential for energy generation, whereas the specific E_out_ array for the cell area is observed at 0.0001. The inverter efficiency, which averages 0.9786, demonstrates the system’s efficiency in converting generated energy. The worldwide incident on the collector plane, which averaged 1681 kWh/m^2^, demonstrates the abundance of solar radiation accessible for energy production. Throughout the year, the sky diffuse incident in the collection plane averaged 459.7, emphasizing diffuse radiation contributions. The Incident Angle Modifier (IAM) value for global radiation is 0.9810, indicating that the system is adaptable to different sun angles, but the IAM factor for circumsolar radiation is 1, indicating that optimal collection from direct sunlight is achieved. With a soiling factor of 0.9000, the system’s cleanliness is critical to its functioning. During operation, the average module temperature is 35.57 degrees Celsius, which has an impact on total efficiency. Finally, the array runs for an average of 3910 h every year, evenly spreading energy output across the months. The dynamic behavior of the average module temperature during system operation is depicted in (f analysis takes a multidimensional approach, taking into account many elements and trends. The impact of an effective global correction factor on module temperature is shown in Figure (a), emphasizing the relevance of this parameter in system performance. We investigate how the number of occurrences affects temperature patterns in subfigure (b), revealing light on the temporal aspects of system operation. (c) and (d) shed light on the relationship between useful system energy and global incident energy, as well as the proportion of useful system energy in the overall context. These visualizations, when combined, provide a full view of module temperature dynamics and lead to a better understanding of system behavior during operation. These insights provide a comprehensive picture of the system’s performance and efficiency. Figure 17 shows the behavior of average module temperature during operation with (a) effective global correction factor, (b) number of occurrences, (c) useful system energy vs. global incident energy, and (d) percentage of useful system energy.

**Table 5:** Balances and main results

Month	GlobalHor (kWh/m^2^)	DiffHor (kWh/m^2^)	T_Amb (°C)	GlobInc (kWh/m^2^)	GlobEff (kWh/m^2^)	EArray (kWh)	E_Grid (kWh)	E_User (kWh)	E_Solar (kWh)	EFrGrid (kWh)
January	76.7	35.34	13.93	113.2	98.3	783,412	783,233	46,342	44,876	1466
February	81.6	39.53	15.94	107.5	95.5	766,076	702,866	43,556	42,494	1062
March	120.4	71.46	19.80	130.6	115.1	911,558	840,315	48,223	48,000	223
April	157.8	76.56	24.11	166.1	146.1	1,141,367	1,066,917	46,667	46,464	201
May	186.1	92.52	29.15	185.6	163.3	1,251,381	1,172,997	48,223	48,041	182
June	186.7	88.69	33.09	182.1	160.4	1,224,847	1,148,615	46,667	46,495	172
July	180.7	96.75	31.72	177.7	156.3	1,200,860	1,123,818	48,223	48,041	182
August	162.5	97.77	29.26	166.1	146.2	1,128,772	1,053,502	48,223	48,025	198
September	151.1	69.33	27.14	167.4	147.4	1,138,505	1,064,171	46,667	46,463	205
October	122.1	58.44	23.87	155.9	138.3	1,076,864	1,001,669	48,223	47,938	284
November	80.0	43.03	16.94	117.2	104.2	827,514	759,825	46,667	45,924	744
December	74.3	32.62	13.27	124.9	111.4	892,142	821,764	48,223	46,426	1796
Year	1580	802.04	23.1	1794.3	1582.5	12,343,298	11,539,692	565,904	559,187	6715

### Performance coefficients

**Fig. 18:**
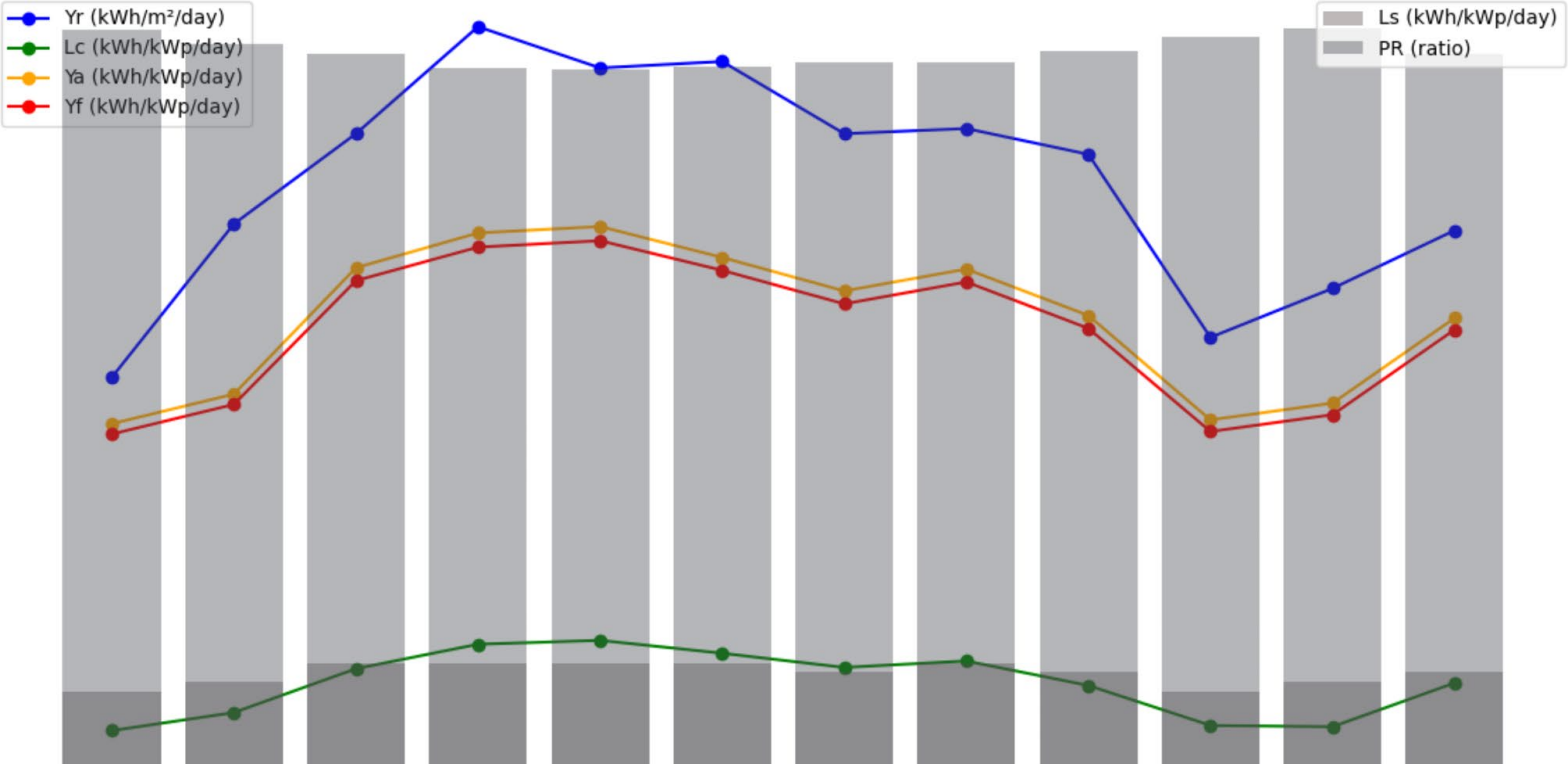
Trends of important performance coefficients for our solar power plant at NUST.

The information shown here indicates the monthly performance coefficients for a solar power system over a range of factors. When the trends are examined (Fig. 18), it is clear that the solar irradiance, denoted by Yr (kWh/m^2^/day), normally rises from February to June, indicating greater solar energy availability during the spring and early summer months. In contrast, it falls from July to December, with the lowest values recorded in December, which corresponds to the winter season. The Lc (kWh/kWp/day) numbers, which measure energy yield per unit installed capacity, follow a similar pattern, with the highest values occurring in May and June and the lowest in December. Similarly, the Ya (kWh/kWp/day) numbers, which indicate the actual energy production efficiency of the solar panels, show a seasonal tendency. Ls (kWh/kWp/day), on the other hand, represents the losses caused by shade or other causes. With minor deviations, these values remain largely stable throughout the year. Yf (kWh/kWp/day) represents energy delivered to the grid and has a similar trend to Ya, with the highest values in May and June. The variables Lcr (ratio), Lsr (ratio), and PR (ratio) represent various system performance ratios. Throughout the year, they remain rather steady. Overall, the data indicates that the solar power system is most efficient throughout the spring and early summer months, with efficiency decreasing over the winter season due to reduced solar irradiation and shorter daylight hours. Daily irradiance (Yr) averages 4.88 kWh/m^2^, with an energy yield (Lc) of 1.01 kWh/kWp. The average energy available for capture (Ya) is 3.77 kWh/kWp, and shading losses (Ls) are 0.10 kWh/kWp. The total energy production (Yf) is 3.88 kWh/kWp. Loss ratios (Lcr and Lsr) remain low at 0.211 and 0.019, assuring dependability. The average performance ratio (‘PR’) is 0.767 or 76.7%, indicating efficient energy conversion.

### Normalized productions and reference incident energy

**Fig. 19:**
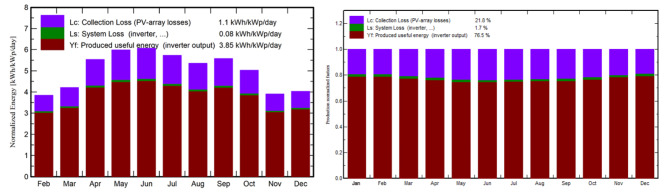
Comprehensive assessment of solar PV system performance, losses, and efficiency metrics.

The LC value (Loss of Collector) for the PV array is 1.1 kWh/kWp/day, whereas the LS value (Loss of System) for the entire system, including inverters and other components, is 0.08 kWh/kWp/day. In addition to these data, the YF value (Yield Factor), which includes inverter output, is 3.85 kWh/kWp/day. These data are important in evaluating the overall performance and efficiency of the solar PV system, as shown in (Fig. 19). It’s worth mentioning that the system’s average reference incident energy is 5.033 kWh/m^2^/day, which serves as a baseline for assessing its energy generation capabilities and system losses. These insights aid in optimizing the system’s performance and energy production.

### Loss diagram

**Fig. 20:**
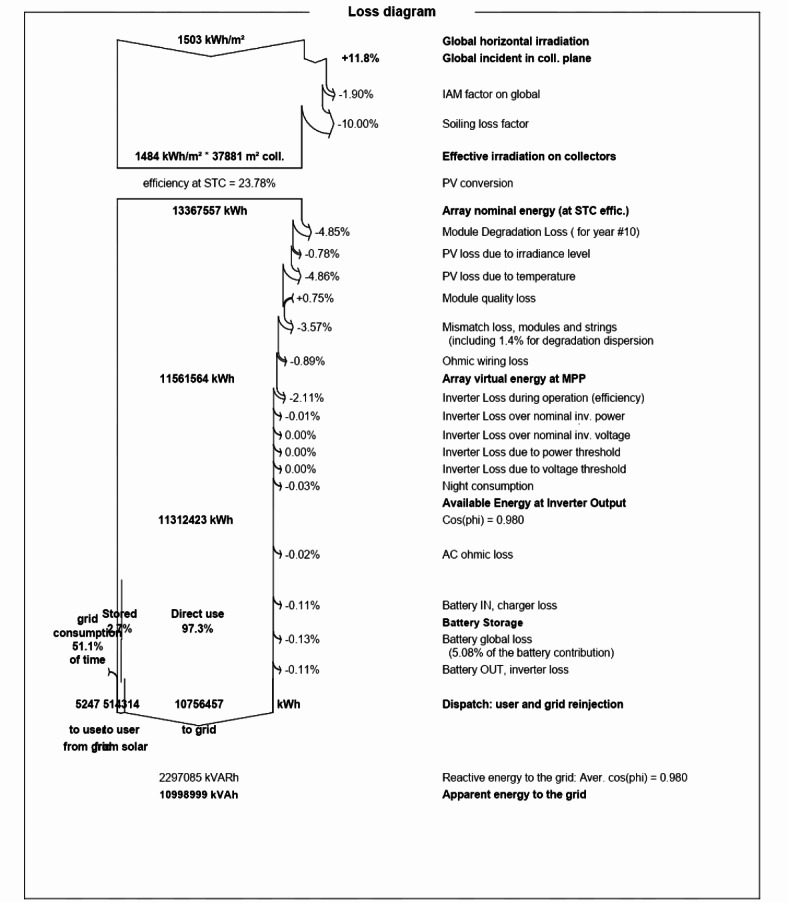
Detailed loss diagram for complete system.

The global horizontal irradiance measures 1503 kW h/m^2^, while the effective irradiation on the collector plane is 1484 kW h/m^2^, as a result, there is an 11.8% loss in energy. The solar energy incident on the solar panels is converted into electrical energy. After the PV conversion process, the nominal array energy amounts to 13,367,557 kWh. The PV array achieves an efficiency of 21.42% under standard test conditions (STC). The array’s virtual energy obtained is 11,561,564 kWh with 4.85% module degradation loss, 0.78% PV loss due to irradiance level, 4.86% PV loss due to temperature, 0.75% module quality loss, 3.57% mismatch loss including the degradation dispersion, and 0.89% ohmic wiring loss. Factoring in inverter losses, the final available energy at the inverter output is 11,312,423 kWh and 10,756,458 kWh ready for the grid (Fig. 20).

### Variations in energy export vs. consumption on various days

Some of the power generated by the solar plant is used for internal purposes. The power output varies daily depending on radiation levels, but the consumption for the plant’s load remains constant, day or night. The goal is to have a large gap between energy export and consumption. The solar plant utilizes 140 kWh during the day and 230 kWh at night. The notion of net metering is used to calculate the electricity utilized by internal utilities as well as the power exported to the grid. Furthermore, power usage is controlled by solar radiation, and the facility records daily and nocturnal use. Power generation may be halted due to poor weather circumstances such as heavy rainfall or low radiation.

### Performance ratio (PR) and capacity utilization factor (CUF)

**Fig. 21:**
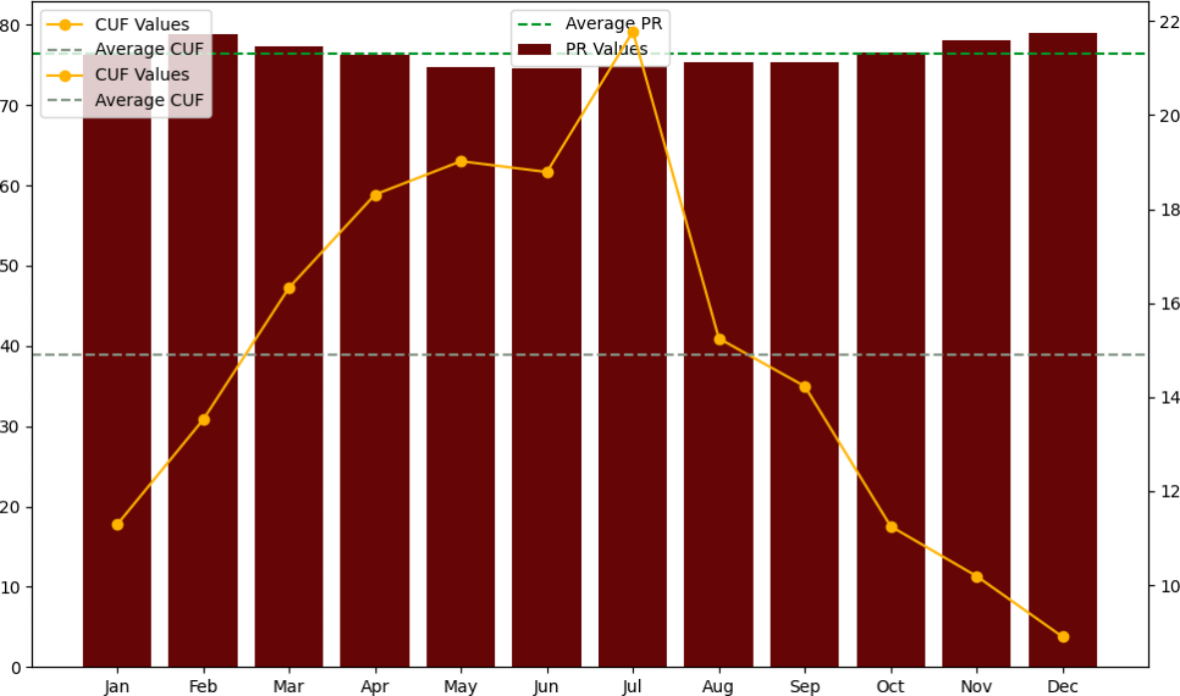
Average performance ratio (PR) and capacity utilization factor (CUF).

The “Performance Ratio” is calculated by dividing the final yield by the reference yield. It serves as a comparison metric, indicating the plant’s actual output relative to its potential output, considering various factors such as irradiation, panel temperature, grid availability, aperture area size, nominal power output, and temperature correction values. The performance ratio provides valuable insights into the efficiency and effectiveness of the solar PV plant, reflecting how well it utilizes available solar resources and performs under real-world conditions^76^. The annual average value of the Performance Ratio (PR) is approximately 76.18%. The highest PR value recorded was 79.10% in December, while the lowest PR value was 74.6% in June. By analyzing the PR values, system malfunctions can be identified and deducted. The data displays a solar power system’s monthly performance ratio (PR) and capacity utilization factors (CUF) over the course of a year. In terms of PR, the system’s performance is reasonably consistent, with PR values ranging from 74.6% in June to 79.0% in November, with an average PR of around 76.6%. This demonstrates that solar irradiance is converted into electricity with consistent efficiency throughout the year. CUF readings, on the other hand, show a seasonal tendency, with the maximum CUF of 21.78% in July and the lowest of 8.90% in December, reflecting fluctuations in energy generation caused by weather and solar angles. The CUF is roughly 15.09% on an annual basis, illustrating the system’s ability to make effective use of its installed capacity, with higher usage during peak solar months (Fig. 21). These PR and CUF insights aid in evaluating the overall performance and energy production efficiency of the solar power system.

### Comparison with other studies

In a comprehensive global study, solar PV systems were tested across varied climate conditions, with Pakistan’s semi-arid climate standing out as a good choice (Table 6). The 11.5 MW solar power plant in Pakistan has an excellent Performance Ratio (PR) of 76.18% and a Capacity Factor (CF) of 15.09%. This exceptional combination produces a Reference Yield of around 2,155,442 kWh, proving Pakistan’s proficiency in solar energy usage. Pakistan’s success has been mirrored in other countries, including as Kuwait, Saudi Arabia, and Chile, each of which has its unique climate and PV technology. Kuwait’s subtropical climate benefits from Poly-Si technology, resulting in a Reference Yield of 3,256,122 kWh. Saudi Arabia obtains an 88.29% PR using Mono-Si technology in its arid desert circumstances, giving in a Reference Yield of 2,648,800 kWh. Meanwhile, Mono-Si technology thrives in Chile’s arid climate, producing an incredible Reference Yield of 2,107,040 kWh. These findings demonstrate PV technologies’ adaptability to a variety of climates, as well as the global significance of solar energy as a sustainable power source. While each area has its own set of challenges and opportunities, these studies demonstrate solar energy’s ability to meet the world’s energy needs while also supporting environmental sustainability. Pakistan’s outstanding success serves as an example for other countries seeking to fully exploit the potential of solar energy.

**Table 6:** Comparative performance of solar PV technologies in varied climate conditions.

Location	Climate condition	Studied technology	Size (MW)	PR (%)	CF (%)	Reference yield (kWh)	References
South Africa	Arid	Thin film	7	76.60	16.90	1,459,088	^76^
Spain	Sub-tropical	Mono-Si	4	78.20	17.60	210,752	^51^
Australia	Arid	Poly-Si	8	85.50	20.40	1,741,920	^77^
Sweden	Sub-tropical	Poly-Si	1.2	71.40	16.30	153,810	^50^
Greece	Temperature	Poly-Si	0.1713	67.40	15.30	54,366	^78^
Italy	Temperature	Mono-Si	0.96	84.40	15.66	201,251	^79^
Japan	Humid Sub-Tropical	CIGS	5	77.87	17.70	1,374,570	^80^
China	Temperature	Mono-Si	12	79.60	18.90	2,415,360	^81^
Nigeria	Tropical	Poly-Si	7.6	78.90	17.10	1,313,748	^82^
Egypt	Arid Desert	Mono-Si	12.5	86.50	19.40	2,602,875	^83^
Argentina	Temperature	Poly-Si	5.2	80.80	18.43	1,542,080	^84^
India	Desert, Semiarid	Mono-Si	3	70	15.60	129,144	^85^
Kuwait	Arid Subtropical	Poly-Si	5.6	80.20	20.66	3,822,430	^86^
Pakistan	Semi-Arid	Thin film	8.3	75.60	16.40	1,272,792	^47^
Chile	Arid	Mono-Si	11.8	86.80	19.60	2,107,040	^87^
Canada	Temperature	Poly-Si	3.5	74.90	16.80	632,124	^88^
Brazil	Tropical	CIGS	6.5	82.30	18.50	1,217,585	^89^
Saudi Arabia	Arid Desert	Mono-Si	15	88.29	20.20	2,648,800	^90^
France	Temperature	CIGS	7.7	77.30	16.80	1,123,368	^91^
UAE	Arid Desert	Poly-Si	8.5	85.10	20.02	1,454,340	^92^
Mexico	Arid	Thin film	9.2	74.20	16.10	1,077,712	^93^
Russia	Cold	CIGS	4.8	72.60	15.80	809,280	^94^
Mauritania	Arid	Micro morph-Si	0.9543	67.90	17.75	20,864	^95^
Pakistan	Semi-Arid	Mono-Si	8.79	76.18	14.94	3,256,122	This research

## Carbon footprint reduction and environmental impact

The completion of an 8.78 MW solar power plant at the National University of Sciences and Technology (NUST) is not only a big step toward clean energy, but it is also a significant contributor to reducing carbon footprint. The carbon balancing is predicted to save 75,478.60 tons of CO_2_ emissions over the course of 18 years, based on calculations performed using PVsyst software, which estimates the environmental impact of solar power installations. This equates to an astounding 4,193.259 tons of CO_2_ reduction each year, underlining its environmental sustainability even further. Furthermore, the carbon footprint reduction rate per installed kWp is 8.579 tons of CO_2_ and the annual reduction rate is 0.477 tons of CO_2_ per kWp.

## Cost analysis of solar power plant

**Fig. 22:**
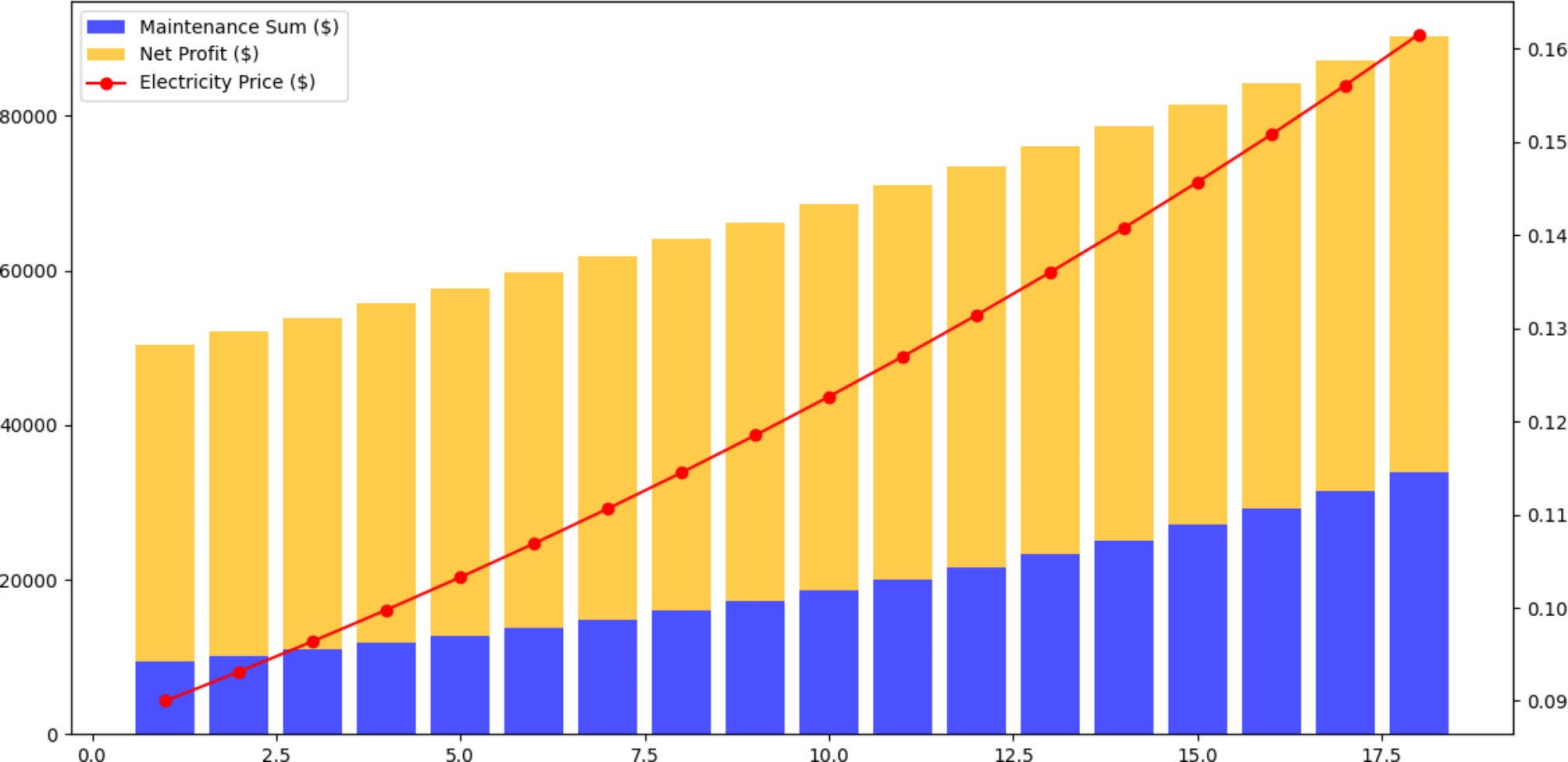
Financial analysis of solar PV system over 18 years.

**Fig. 23:**
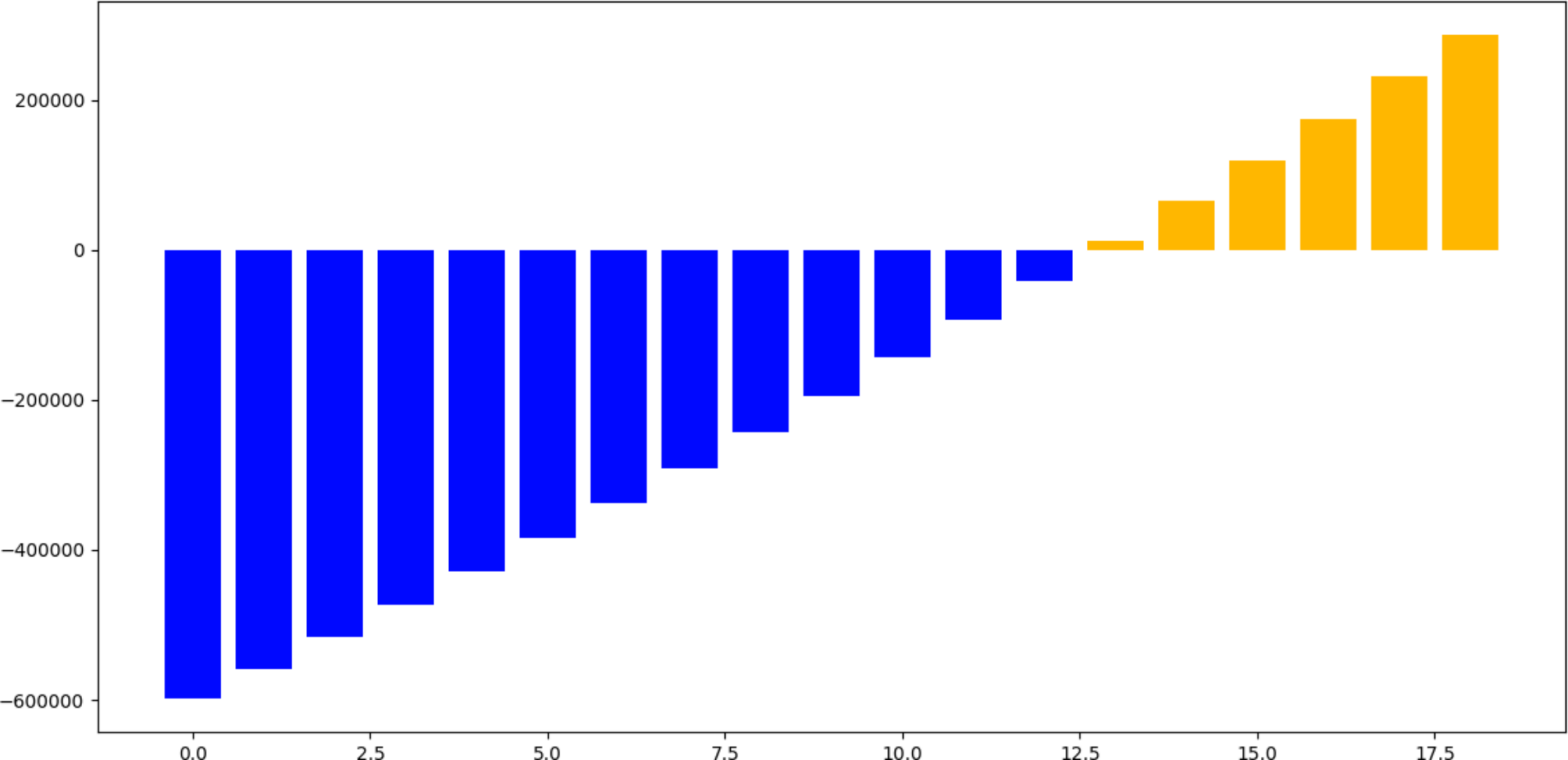
Cash flow analysis for solar PV system over 18 years.

Our 18-year cost analysis of the NUST power plant project provides useful insights into the project’s financial dynamics. Maintenance expenditures have risen steadily throughout the years, as shown in Fig. 22. According to statistical data, maintenance expenses climb at an annual pace of around 12.2% on average. This graph represents the usual operational costs associated with the operation of a large-scale solar power plant. A cash flow and payback period can be calculated using the known cost of the entire solar system retrofit and is illustrated that within 12 years of the installation, the cash flow will be positive in net profit (Fig. 23). The following assumptions were considered:

- No Financial Loans: It is important to highlight that no financial loans were taken to cover the initial investment in the solar system retrofit. As a result, no connected interest rates affect the financial analysis.
- Exclusion of Depreciation: For the sake of this analysis, the depreciation of the investment has been ignored, focusing simply on the project’s initial and ongoing financial considerations.
- Considerations for Inflation: A yearly inflation rate of 8.26% has been considered in the calculations. This compensates for anticipated buying power depreciation over time, resulting in a more realistic projection of future cash flows.
- We stressed financial resiliency considering unplanned maintenance expenditures, system downtime, and changing energy pricing or regulations.
- Environmental and social impact: We evaluated environmental and social benefits, such as lower carbon emissions and community impact, in our analysis.
- Scalability: We investigated the possibility of future growth for higher energy generation and revenue.
- Alternative Investments: We assessed the risk-return profiles of our project and compared them to traditional investments.
- Power Price Escalation: To account for the changing energy landscape, the estimates include a 1.6% yearly increase in power prices.
- Maintenance Expenses: To sustain operating efficiency, the system’s maintenance expenses have been projected to be 12.2% of the entire initial CAPEX (Capital Expenditure) cost.
- Power Tariff Benchmark: The home power tariff in Pakistan is $0.09 per kWh as of 2023.
- Sensitivity Analysis: We looked at how changes in variables, such as electricity prices or maintenance expenses, affected project outcomes.
- Long-Term Sustainability: We looked beyond repayment to assess the project’s long-term viability.Conclusion.

An 8.75 MW grid-connected Photovoltaic (PV) system has been proposed for The National University of Sciences and Technology (NUST) in Islamabad, Pakistan, in response to the important worldwide issue of climate change. This strategic location, rich in solar resources, makes a compelling case for harnessing clean and sustainable energy. The following are the important themes and findings from our extensive research:

- Abundant Solar Resources: Islamabad has a daily solar irradiation of 5.89 kWh/m^2^ and a solar percentage of 98.99%. This makes it an excellent position for capturing solar energy.
- Environmental Impact: Our PV system is expected to avoid 20,000 tons of CO_2_ emissions over a five-year period, which is equivalent to planting 348,754 teak trees. This emphasizes the system’s enormous environmental advantages.
- Electricity Generation at a Low Cost: Our PV system generates electricity at a low cost of 0.0141 US $/kWh, making it a financially feasible and sustainable alternative to traditional energy sources.
- Inverter Efficiency: For the project, the Huawei Technologies SUN2000-50KTL-M3-380 V, H inverter with an efficiency of 98.26% was chosen, ensuring optimal energy conversion.
- Adaptive Tilt Strategy: Our research found that using seasonal tilt angles increased solar irradiation by 36.31%, improving overall energy efficiency.
- High-Efficiency Panels: For their efficiency and longevity, monocrystalline silicon Longi_LR5-72_HTH_600M panels with a power output of 600 Wp were chosen.
- Performance Indicators: PR values have regularly ranged from 74.6 to 79.0%, with an average of around 76.6%. The annual average of Capacity Utilization Factor (CUF) values is 15.09%.
- Net Metering: A net metering system has been deployed to calculate electricity utilization for internal utilities and power export to the grid, ensuring optimal energy management.
- Our findings not only illustrate the enormous potential of grid-connected renewable energy, but also provide useful insights for future sustainable energy programs.

In conclusion, our PV system at NUST, Islamabad, is a promising solution for combating climate change and promoting sustainability. It serves as a paradigm for clean energy adoption by using copious solar resources, innovative technology, and cost-efficiency. This project lays the groundwork for a more environmentally friendly and sustainable future.

## Data Availability

The datasets generated and/or analyzed during the current study are not publicly available due to concerns about potential cloning or misuse but are available from the corresponding author on reasonable request.
